# A halogen bonding BODIPY-appended aza-crown ether for selective optical sensing of inorganic and organic ion-pair species

**DOI:** 10.1039/d5sc05033b

**Published:** 2025-09-08

**Authors:** Jamie T. Wilmore, Andrew J. Taylor, Igor Marques, Vítor Félix, Paul D. Beer

**Affiliations:** a Department of Chemistry, Chemistry Research Laboratory, University of Oxford Mansfield Road Oxford OX1 3TA UK paul.beer@chem.ox.ac.uk; b Department of Chemistry, CICECO–Aveiro Institute of Materials, University of Aveiro 3810-193 Aveiro Portugal

## Abstract

A novel ion-pair receptor, consisting of a diaza-18-crown-6 cation binding motif and a BODIPY-appended halogen bonding optical anion-sensing motif, is prepared and shown to be a potent receptor for inorganic and organic ion-pairs. Comprehensive optical emission and ^1^H NMR spectroscopic titration studies, in combination with computational investigations, demonstrate that the receptor exhibits significant selectivity for the ion-pair guest species K^+^/Cl^−^ over other alkali metal halide ion-pairs, and the neurotransmitter salt dopamine hydrochloride over other phenethylamine hydrochloride derivatives. Notably, the specific host–guest three-dimensional binding mode, resulting from the simultaneous participation of the heteroditopic receptor's multiple proximal convergent binding motifs, dictates the observed inorganic and organic ion-pair selectivity trends, representing a potent biomimetic strategy for the design of optical sensors for a range of biologically-relevant ion-pair analytes.

## Abstract 2

An optically-responsive ion-pair receptor combining aza-crown cation and halogen bonding anion binding sites selectively binds dopamine·HCl through the formation of a convergent chloride binding site between the receptor and co-bound dopamine cation.

## Graphical abstract

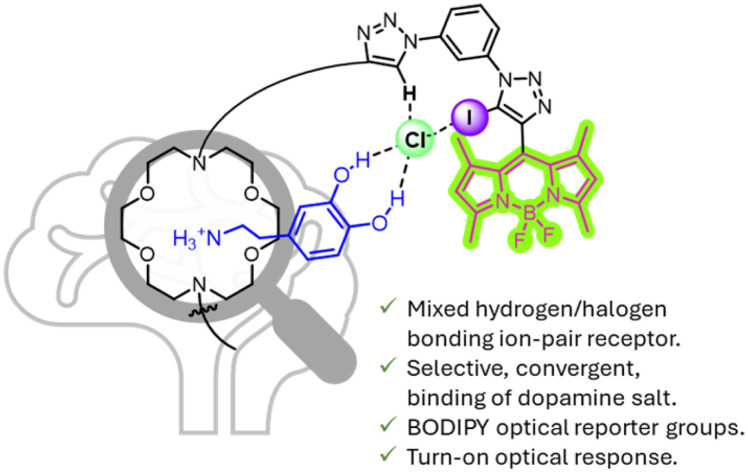

## Introduction

Within the field of supramolecular host–guest recognition and sensing, ion-pair recognition – the simultaneous selective binding and recognition of both a cation and anion – has garnered increasing interest, not least due to the field's wide-ranging potential applications. These include precious metal salt extraction,^1^ lithium salt recovery for sustainable battery development,^2–4^ the detection and remediation of environmental pollutants in watercourses,^5^ and transmembrane transport of biomedically relevant ions for healthcare applications.^6–8^

A principal challenge in the recognition of ion-pairs is the significant energetic penalty required to separate an ion from its counter-ion, particularly in organic media. To overcome this challenge, throughout the past few decades a variety of heteroditopic receptors have been constructed,^9–11^ wherein the cation and anion are simultaneously bound at distinct binding sites, exploiting favourable electrostatic and allosteric effects to increase binding selectivity and affinity.^12–15^ Furthermore, in recent years, we and others have demonstrated that the incorporation of sigma–hole interactions, such as halogen bonding (XB) and chalcogen bonding, into the anion binding motifs of heteroditopic receptor designs offers significantly improved ion-pair recognition capability and performance.^16–18^ In particular, previous reports have combined XB anion binding motifs alongside a range of cation binding sites, including ethylene glycol chains,^19–21^ phenanthroline groups,^2^ triazoles,^22^ and squaramides,^23^ to enhance binding of both alkali metal and ammonium halide ion pairs.^12,24–26^

In addition to the strong and selective recognition and binding of an ion-pair, the sensing of ion-pair substrates is of ever-increasing interest. This necessitates the integration of a reporting transducer motif into a heteroditopic structural host framework. While various optical and electrochemical systems have been reported,^27–29^ the BODIPY fluorophore is an attractive optical responsory group to employ due to its strong absorption and high quantum yield,^30^ and has previously been demonstrated to exhibit strong optical responses to monotopic host–guest binding events.^31–33^

**Fig. 1:**
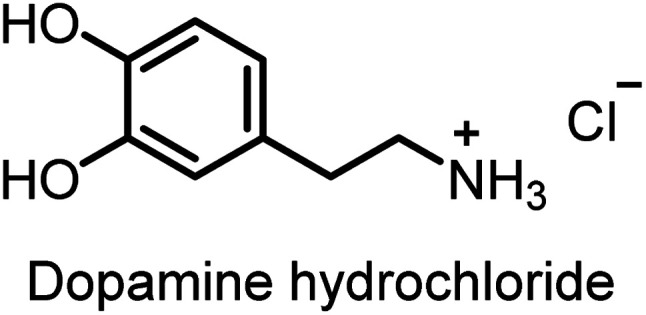
Structure of dopamine hydrochloride.

Dopamine (Fig. 1) is a key neuromodulator, the mis-regulation of which is linked to a range of pathologies including Parkinson's and Alzheimer's diseases.^34^ As a result of their biological function, dopamine and related phenethylamine salts pose a significant environmental risk, with residues of these species being detected in wastewater discharges from industrial and healthcare facilities,^35,36^ leading to a clear imperative for the development of systems capable of the selective sensing and remediation of these organic ammonium salts.

**Fig. 2:**
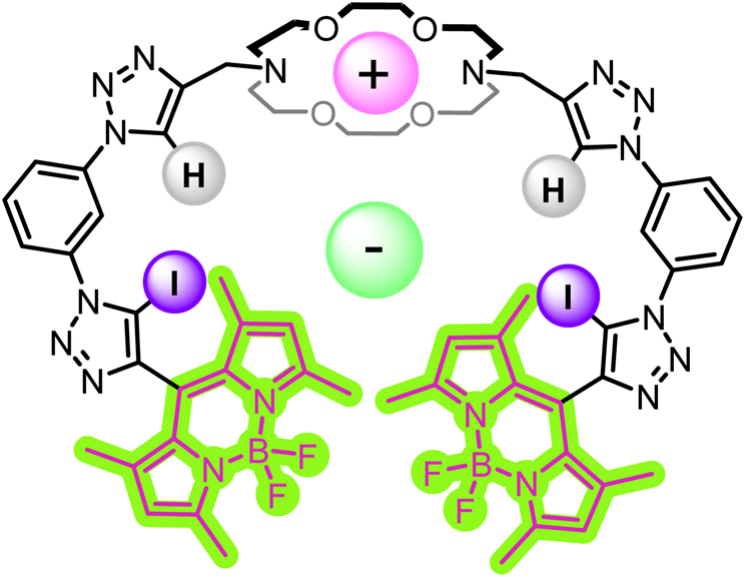
Schematic depiction of an optical response to binding of an ion-pair by heteroditopic sensor 3.

Herein, we report ion-pair receptor 3, containing a diaza-18-crown-6 cation binding moiety, appended with mixed XB/HB iodo- and proto-triazole anion binding motifs and BODIPY fluorophores for the optical sensing of a range of inorganic alkali metal halide and organic ammonium halide ion-pairs in competitive organic solvent mixtures (Fig. 2). Importantly, of the alkali metal chloride salts, heteroditopic receptor 3 displays selective optical sensing of KCl, while dopamine·HCl is sensed selectively amongst the phenethylamine salts studied.

## Results and discussion

### Design and synthesis of XB BODIPY-appended ion-pair sensor

**Scheme 1:**
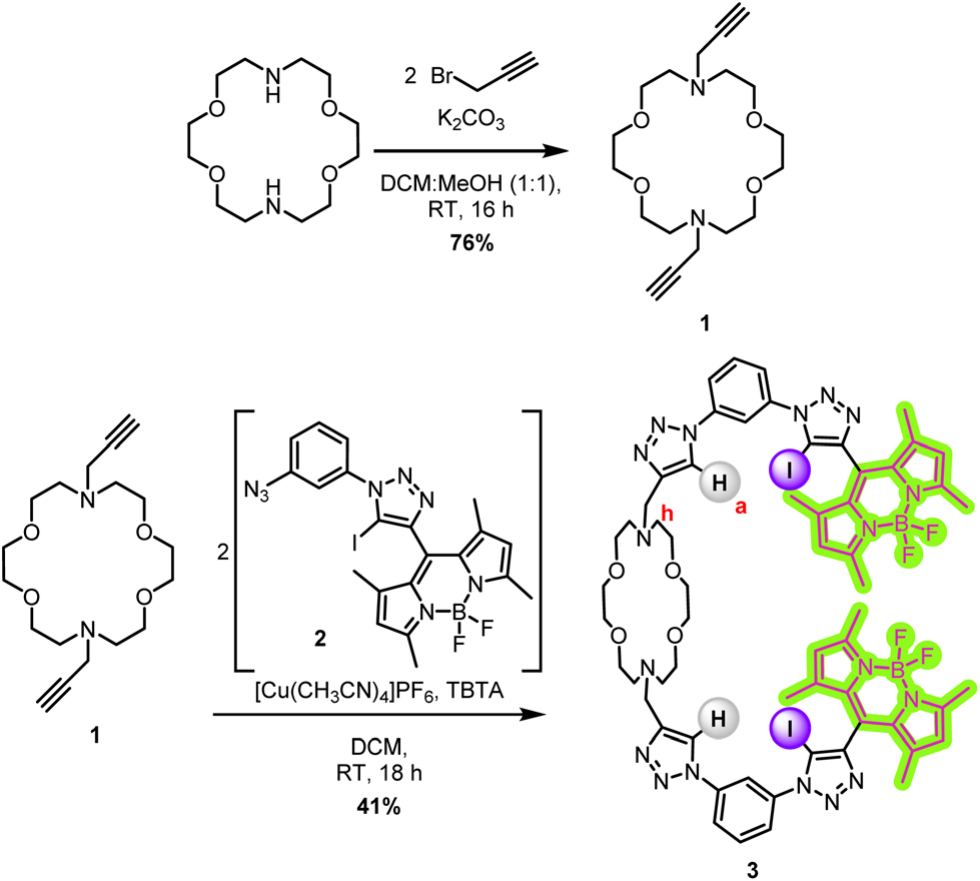
Synthesis of sensor 3*via* a CuAAC methodology.

The target ion-pair sensor features a diaza-crown ether cation binding macrocycle, decorated with two bidentate mixed XB/HB 1,3-bis-(iodo-)triazole benzene anion binding sites, each respectively appended with a BODIPY fluorescent reporter group. The BODIPY-appended (iodo-)triazole anion binding site has previously been demonstrated to be a potent anion sensing motif,^31,37^ and it was anticipated that the combination of this motif with the diaza-crown ether cation binding unit would allow for positively cooperative ion-pair binding, due to the close proximity of the concomitantly bound components of the ion-pair and consequent electrostatic attraction between them. The target sensor was synthesised *via* a copper(i)-catalysed azide–alkyne cycloaddition (CuAAC) methodology (Scheme 1).

The diaza-crown ether bis-alkyne 1 was synthesised from diaza-18-crown-6 by reaction with 2 equivalents of propargyl bromide in the presence of K_2_CO_3_. Purification by alumina column chromatography gave the intermediate bis-alkyne 1 as a colourless wax in 76% yield.‡‡Synthetic attempts *via* a wide variety of methods to prepare the iodo-alkyne analogue of 1 failed. The BODIPY-containing azide fragment 2 was prepared according to a previously published procedure.^31^ The diaza-crown ether bis-alkyne 1 was reacted with two equivalents of the azide fragment 2 under CuAAC conditions to afford the target XB BODIPY-diaza-crown ether functionalised heteroditopic receptor 3 in an isolated 41% yield following purification *via* alumina column chromatography. Sensor 3 was characterised by ^1^H and ^13^C NMR, UV-vis spectroscopy and HR-ESI-MS (see SI, Sections S2 and S3).

### Cation fluorescence sensing, ^1^H NMR binding, and computational modelling studies

**Fig. 3:**
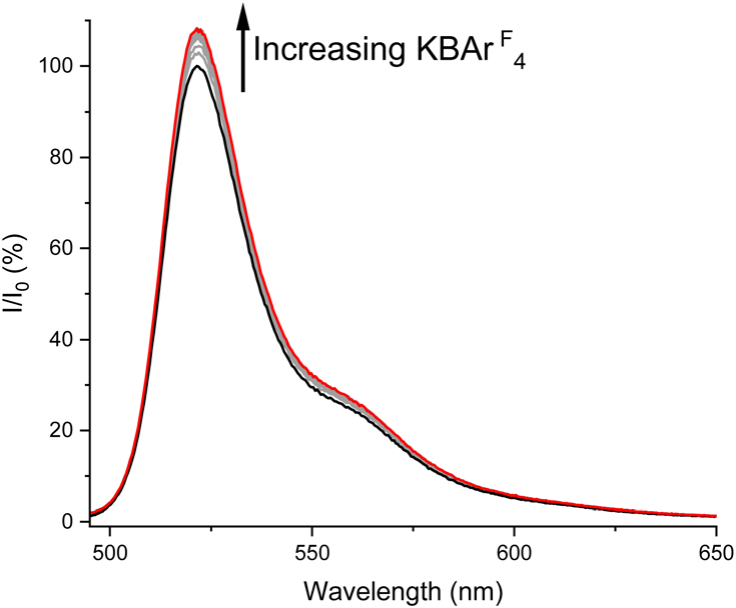
Stacked emission spectra of 3 with increasing concentrations of KBAr^F^_4_ (up to a maximum of 2.86 mM) (1 μM, acetonitrile/methanol 1 : 1 (v/v), 298 K).

Initially, the fluorescence sensing behaviour of sensor 3 to sodium and potassium cation binding was assessed through fluorescence titration experiments. In these experiments, aliquots of Na^+^ and K^+^ cations as their BAr^F^_4_^−^ salts were added to an acetonitrile/methanol 1 : 1 (v/v) solution of 3 and the emission spectrum monitored. A modest turn-on increase in BODIPY emission intensity of 8% at 521 nm upon addition of KBAr^F^_4_ was observed (Fig. 3). As expected, the binding affinity between the diaza-crown ether and the K^+^ cation was strong, and an association constant, *K*_a_, of 31 300 M^−1^ was calculated by global fitting of the titration data to a 1 : 1 host–guest stoichiometric binding model using Origin software.^38^ In contrast, addition of NaBAr^F^_4_ led to much smaller (<3%) turn-off changes in the emission spectrum of sensor 3, too small a response to be reliably fitted to a binding model to calculate an association constant (SI, Fig. S5). Importantly, this differing behaviour means that sensor 3 exhibits the ability to discriminate between Na^+^ and K^+^ cations.

**Fig. 4:**
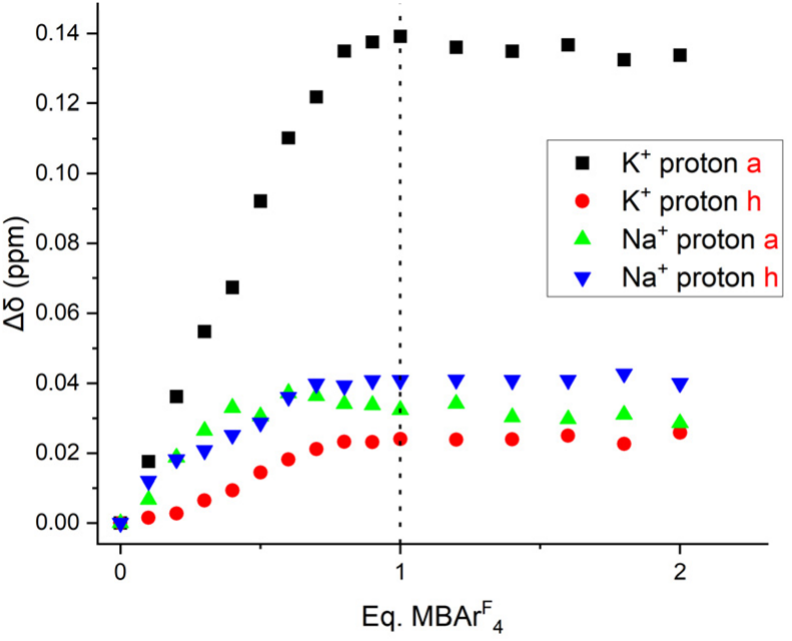
The shift of receptor 3 protons *H*_a_ and *H*_h_ as a function of alkali metal cation concentration (500 MHz, 1 mM, acetone-*d*_6_, 298 K).

**Fig. 5:**
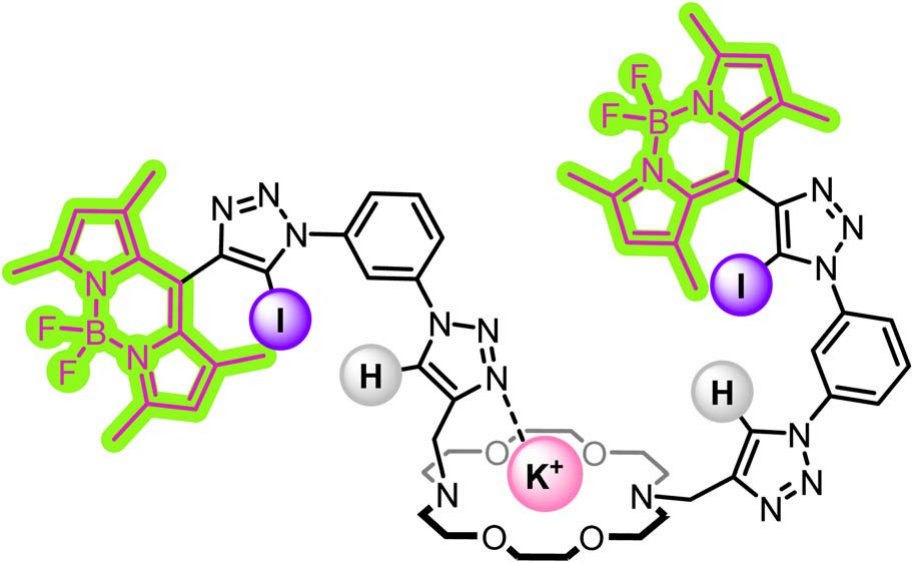
Cartoon displaying the proposed K^+^ binding conformation of sensor 3, with the coordination of one of the triazole groups to the bound K^+^ cation.

To investigate the origin of the contrasting fluorescent emission responses to K^+^ and Na^+^ binding, ^1^H NMR titrations were conducted, also using the alkali metal BAr^F^_4_^−^ salts. Due to the much higher concentrations required for ^1^H NMR experiments, these titrations were conducted in acetone-*d*_6_ for solubility reasons. Addition of both cations caused significant downfield shifts in the aza-crown ether methylene proton resonances, which plateaued after the addition of 1 equivalent of MBAr^F^_4_ in both cases, consistent with the formation of a strong 1 : 1 stoichiometric complex (SI, Fig. S10 and 11).§§The high stability of the K^+^ and Na^+^ complexes with sensor 3 in acetone-*d*_6_ precluded the calculation of a binding constant in this solvent, although it can be reliably estimated to be >10^5^ M^−1^ in both cases. Interestingly, however, significantly different changes in the triazole proton *H*_a_ NMR shift were observed upon the addition of Na^+^ and K^+^ cations (Fig. 4). Upon K^+^ binding, the triazole *H*_a_ resonance shifts significantly downfield, indicating deshielding, which was attributed to coordination of the triazole *N*-atom to the larger K^+^ cation (Fig. 5). In contrast, a far smaller shift was observed when Na^+^ was added, suggesting that the triazole does not coordinate to this smaller cation. This in turn offers a possible explanation for the differing fluorescence changes observed: the coordination of the triazole moiety to the bound K^+^ cation reduces the conformational flexibility of the sensor, increasing its rigidity and therefore reducing the rate of non-radiative decay processes, leading to an increase in fluorescence quantum yield and hence emission intensity.¶¶An alternative pathway for the optical response, PET inhibition, could potentially arise through cation coordination to receptor *N* atoms. We considered the possibility of this mechanism giving rise to the optical response observed upon cation binding, however this would be expected to result in a similar response for binding of both K^+^ and Na^+^ being observed. As this turn-on response is only observed for K^+^ binding, we attribute the effect to the considerable structural rigidification occurring upon K^+^ binding by the triazole *N* atom, as described in the main text.^31,39^ In contrast, no such rigidification of the sensor is induced by Na^+^ cation binding, and hence the fluorescence response is notably smaller. Thus, the selective K^+^ over Na^+^ sensing capability of 3 may be rationalised by the additional triazole binding mode with the larger alkali metal cation producing a different emission response. A similar approach was recently used for selective chloride sensing by UV-vis absorption spectroscopy.^40^

Further structural insights into the conformational binding preferences of 3 upon K^+^ binding were obtained from molecular dynamics (MD) simulations followed by density functional theory (DFT) calculations, as detailed in the SI. The MD simulations in solution revealed that the triazole units can establish one or two interactions with K^+^, while the DFT calculations indicated that the doubly coordinated conformation is the most stable.

### Inorganic ion-pair sensing studies

**Fig. 6:**
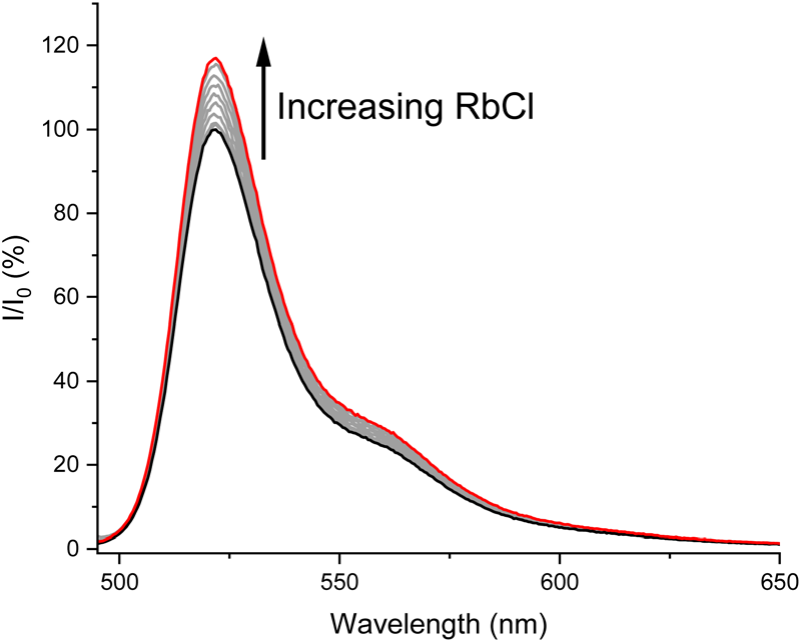
Stacked emission spectra of 3 with increasing concentrations of RbCl (up to a maximum of 7.14 mM) (1 μM, acetonitrile/methanol 1 : 1 (v/v), 298 K).

Following the cation binding studies, attention then turned to assessing the ability of 3 to sense alkali metal halide and ammonium halide ion-pairs. Due to the sufficient solubility of most alkali metal and ammonium halide salts in the competitive acetonitrile/methanol 1 : 1 (v/v) solvent mixture at the low concentration needed for fluorescence spectroscopic studies, direct titration of the alkali metal and ammonium halide salts into a solution of sensor 3 was undertaken.^41^ Addition of KCl, KBr, RbCl, CsCl, NH_4_Cl and NH_4_Br to a solution of 3 in acetonitrile/methanol 1 : 1 (v/v) all caused increases in the emission intensity, attributed to rigidification of the heteroditopic host structure and the restriction of rotation about the *meso* BODIPY bond upon ion-pair binding (Fig. 6).^29^ In a similar manner to the K^+^ cation binding study, binding isotherms for each of the titrations were globally fitted to a host–guest binding model, revealing a 1 : 1 host–guest binding stoichiometry and enabling the determination of apparent association constants (*K*_app_)^42^ of the sensor inorganic ion-pair complexes, shown in Table 1.||||This method assumes strong near quantitative binding of the cation to the ion-pair sensor and that, over the course of the titration, the anion binds to the cationic sensor-M^+^ complex. Given the very high binding constant measured for K^+^ and the likely positive cooperativity between cation and anion binding, we believe this is a reasonable assumption which allows relative differences in association affinity to be assessed. In addition, titrations were also conducted with Cl^−^ and Br^−^, using the non-coordinating tetra-*n*-butylammonium (TBA^+^) counter-ion, in order to assess the degree of cooperativity in the ion-pair binding process for sensor 3.

**Table 1:** Apparent association constants *K*_app_^a^ (M^−1^) of sensor 3

Ion-pair	*K*_app_ (anion) (M^−1^)
KCl	3200
RbCl	970
CsCl	780
NH_4_Cl	1020
KBr	2890
NH_4_Br	550
TBACl	130
TBABr	110

a Determined in acetonitrile/methanol 1 : 1 (v/v) at 298 K by global fitting of fluorescence isotherms to a 1 : 1 host–guest stoichiometric binding model and errors <15%.

Notably, as anticipated the apparent halide anion affinities of the ion-pair complexes were significantly higher in the presence of a diaza-crown ether co-bound coordinating alkali metal or ammonium cation – with fitting of the Cl^−^ anion binding to a 1 : 1 host–guest stoichiometric binding model revealing an impressive 25-fold increase in binding affinity upon changing the counter-cation from TBA^+^ to K^+^. This was attributed to both an electrostatic mechanism of positive cooperativity and also polarisation of the hydrogen-bonding protic triazole and potentially halogen bond donor iodo-triazole moieties for anion binding *via* coordination to the co-bound cation. A similar magnitude of halide anion binding enhancement was observed for the bromide ion-pair complexes with electrostatic and through-bond polarisation mechanisms of cooperativity.^12^ The trend of decreasing Cl^−^*K*_app_ upon descending the group 1 metal cations (*K*_app_(KCl) > *K*_app_(RbCl) > *K*_app_(CsCl)) reflects the strength of the underlying M^+^-diaza-crown complexes, which decrease K^+^ > Rb^+^ > Cs^+^, driven by the size-complementarity of diaza-18-crown-6 for K^+^ over other alkali metal cations.^43^

### Alkyl ammonium–chloride ion-pair sensing studies

**Fig. 7:**
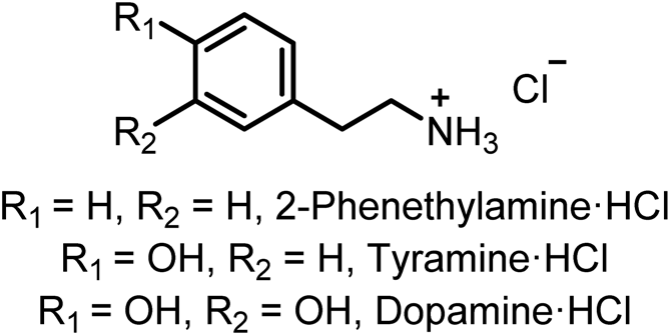
Structures of phenethylamine salt derivatives tested in ion-pair binding studies.

Crown ethers are also known to form complexes with aliphatic alkyl ammonium compounds.^44,45^ Attention therefore turned to investigating the ability of 3 to sense the hydrochloride salt of dopamine, an important neuromodulator, and related phenethylamine salts (Fig. 7). Fluorescence titrations conducted in the same solvent mixture (MeCN : MeOH v/v 1 : 1) resulted in significant emission intensity enhancements in all cases. The results were globally fitted to a 1 : 1 stoichiometric host–guest binding model to determine apparent association constants (*K*_app_) of organic alkyl ammonium ion-pair complexation with sensor 3, shown in Table 2.

**Table 2:** Apparent association constants *K*_app_^a^ (M^−1^) of sensor 3 with phenethylamine-derivative ammonium hydrochloride salts

Ion-pair	*K*_app_ (M^−1^)
Dopamine·HCl	1530
Tyramine·HCl	830
2-Phenethylamine·HCl	810

a Determined in acetonitrile/methanol 1 : 1 (v/v) at 298 K by global fitting of fluorescence isotherms to a 1 : 1 host–guest stoichiometric binding model and errors <16%.

**Fig. 8:**
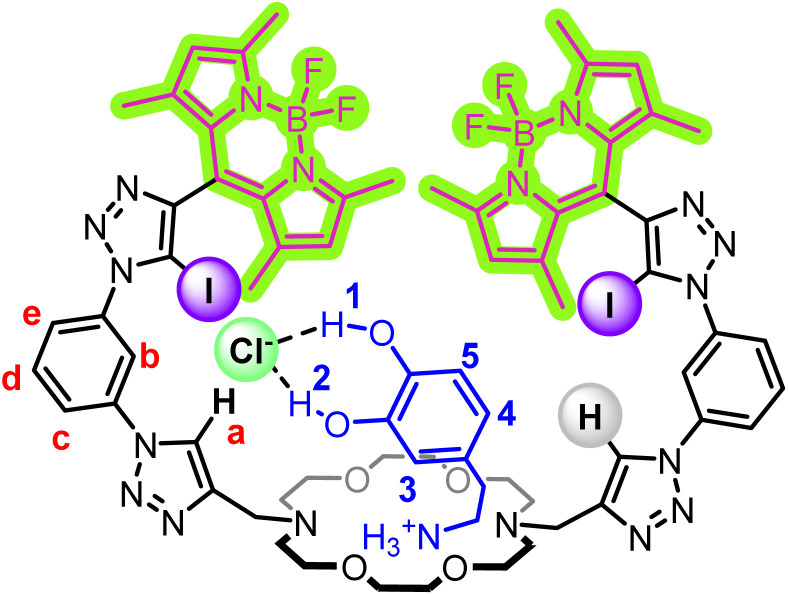
Cartoon displaying a possible dopamine·HCl binding conformation of sensor 3, with the formation of hydrogen bonds between the catechol –OH groups of dopamine and the co-bound Cl^−^ anion.

Pleasingly, sensor 3 demonstrated selectivity for the neurologically-relevant dopamine·HCl ion-pair over related phenethylamine-derivative ammonium salts tested. Taking into account that simple catechol derivatives are known to strongly bind the Cl^−^ anion through chelating hydrogen bonding interactions in competitive, aqueous-containing solvent mixtures,^46^ the significantly increased affinity of the dopamineH^+^-3 complex for Cl^−^ may be attributed to formation of hydrogen bonds between the catechol hydroxyl groups of the diaza-crown ether bound [dopamine + H^+^] cation and the Cl^−^ anion, which act as an additional stabilising interaction concomitant with the receptor's iodo-triazole XB and protic-triazole HB interactions (Fig. 8).

**Fig. 9:**
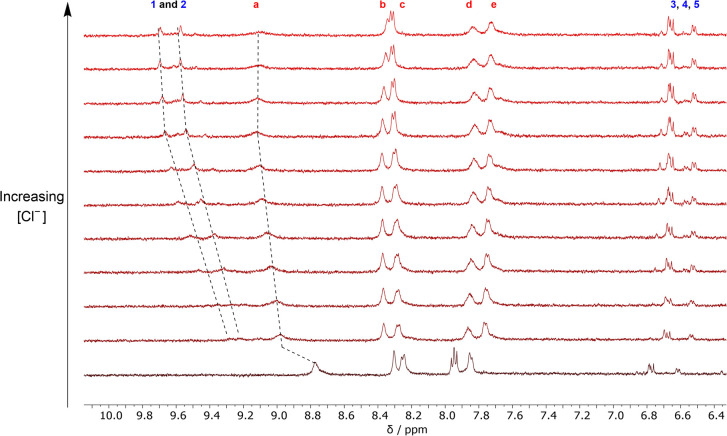
Stacked ^1^H NMR spectra of an equimolar solution of 3 and dopamine·HPF_6_ showing the aromatic region upon increasing concentrations of Cl^−^ (500 MHz, 1 mM, acetone-*d*_6_, 298 K).

In order to probe the existence of these halide-catechol HB interactions further, a ^1^H NMR titration experiment was conducted in acetone-*d*_6_ in which 3 was first complexed with 1 equivalent of dopamine·HPF_6_, followed by the addition of aliquots of TBACl. Upon the addition of dopamine·HPF_6_ (Fig. 9), marked downfield shifts were observed in the receptor diaza-crown ether protons, confirming binding of the ammonium motif within the diaza-crown ether cavity. Importantly, upon subsequent TBACl addition, marked downfield shifts in both 3 triazole *H*_a_ and the dopamine catechol hydroxyl protons were observed, strongly indicating that binding occurs through the proposed binding mode. This binding mode creates a chloride binding site which is highly reminiscent of biotic systems, where HB interactions from multiple components of a host are highly pre-organised to bind a target charged guest in a three-dimensional cavity.^47^

Such additional catechol group stabilising interactions between the components of the tyramine·HCl and 2-phenethylamine·HCl ion-pair are not possible, which rationalises their diminished apparent association constant values (Table 2).

### Ion-pair binding computational studies

**Fig. 10:**
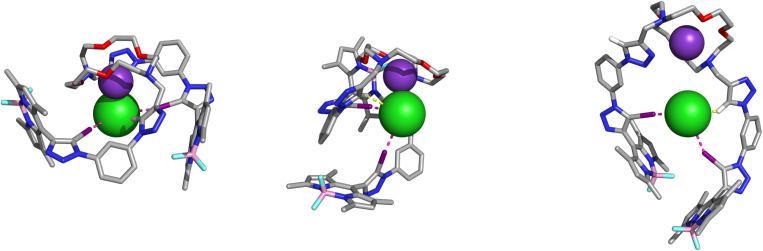
DFT gas-phase optimised structures of 3·KCl in the KCl_α_ (left), KCl_β_ (middle), and KCl_γ_ (right) alternative conformational binding scenarios.

The KCl ion-pair complex of 3 was also explored *via* computational modelling. Given the conformational flexibility of the XB-BODIPY pendant arms observed in the MD simulations with K^+^ (see SI), initial Cl^−^ binding arrangements were obtained from conformational searches using quenched MD simulations (see SI). Two representative conformations featuring putative convergent C_trz_–I⋯Cl^−^ interactions were selected and optimised at the M06-2X/def2-TZVP level, with halogen atoms described with the def2-TZVPD basis set. The optimised structures, named KCl_α_ and KCl_β_, are shown in Fig. 10. In KCl_α_, the XB-BODIPY pendant arms wrap around the KCl ion-pair (contact distance of 2.98 Å), forming two nearly linear XB interactions, while the KCl_β_ XB interactions are less linear, although the dimensions of the K^+^···Cl^−^ and C_trz_–H⋯Cl^−^ contacts remain comparable (Table S3). In KCl_α_, both XB binding units are nearly perpendicular to the ion-pair, whilst in KCl_β_ only one unit is perpendicular and the other is almost diametrically opposite to K^+^. As KCl_α_ is favoured by a Δ*E*_conf_ of 5.6 kcal mol^−1^, subsequent computational analyses focused on this lower-energy binding arrangement (Table S4).

KCl_α_ was further evaluated in an acetonitrile/methanol 1 : 1 (v/v) solvent mixture through three independent 50 ns MD runs, using the extra point of charge approach to describe the XB interactions (see SI). While both XB interactions are preserved throughout the simulation time, the close contact between Cl^−^ and the encapsulated K^+^ cation is lost within the first 300 ps of MD run, due to the quick solvation of the cation by acetonitrile molecules and of the anion by methanol molecules (Fig. S15). KCl_α_ subsequently opened into binding conformations resembling K_β_ (see SI), with a single triazole moiety interacting with K^+^ and the other proto-triazole pointing to the anion for most of the simulation. This conformational rearrangement is depicted in Fig. S16, which illustrates the evolution of the K^+^··· Cl^−^ and K^+^···N_trz_ distances over time. In contrast with the simulations of 3·K^+^ (Fig. S12) in both solvent systems, no coordination events simultaneously involving both triazole rings and K^+^ were observed.

The most prevalent binding conformation was also characterised by DFT calculations in the gas-phase. The optimised structure KCl_γ_ (Fig. 10) shows Cl^−^ at 5.74 Å from K^+^, coordinated by two iodo-triazole binding sites with I⋯Cl^−^ distances of 3.00 and 3.32 Å, the latter being less linear (Table S3). The XB interactions are assisted by a C_trz_-H⋯Cl^−^ short bonding contact, with an H⋯Cl^−^ distance of 2.55 Å from one of the proto-triazole moieties. The other proto-triazole unit binds K^+^ with a K^+^···N_trz_ distance of 2.78 Å.

The XB interactions in both binding scenarios were also characterised by Natural Bond Orbital (NBO) analysis, focusing on the charge transfer interactions between the lone pair orbitals of Cl^−^ and the antibonding orbitals of the C–I bonds (*n*_Cl^−^_→σ*_C–I_, Table S5). In KCl_α_, the Second-order Perturbation Theory (*E*^2^) values are 12.8 and 14.6 kcal mol^−1^, reflecting the comparable XB dimensions. In contrast, in KCl_γ_, the longer XB interaction has an *E*^2^ value of 6.9 kcal mol^−1^, while the shorter one yields 21.1 kcal mol^−1^. Extending this analysis to the C_trz_–H⋯Cl^−^ bonding contacts (*n*_Cl^−^_→σ*_C–H_, Table S5), the *E*^2^ values amount to 2.2 and 4.4 kcal mol^−1^ in KCl_α_ and KCl_γ_, respectively. This analysis indicates that Cl^−^ is primarily recognised by XB interactions and synergistically assisted by HB interactions, albeit in KCl_α_ the contact ion-pair is additionally characterised by an *E*^2^ value of 15.7 kcal mol^−1^ (*n*_Cl^−^_ → n*_K^+^_, Table S5).

The gas-phase 3·KCl complex structures KCl_α_ and KCl_γ_ were re-optimised at the same level of theory in acetonitrile and methanol (PCM solvent model). The impact of both solvents on the computed structures was negligible, namely in the dimensions of XB interactions (Table S6) leading to RMSD values of 0.28 to 0.35 Å between non-hydrogen atoms. KCl_α_ is favoured over KCl_γ_ by a Δ*E*_conf_ of 6.6 kcal mol^−1^ in methanol and 6.5 kcal mol^−1^ in acetonitrile (Table S4). This preference for KCl_α_ is expected given the conformational flexibility of the host, and because contact ion-pair binding is inherently more electrostatically favourable than recognising Cl^−^ distant from K^+^. Moreover, MD simulations suggest that solvent molecules may drive the transition from KCl_α_ to KCl_γ_ upon ion-pair dissociation. Overall, the DFT and MD results support KCl_γ_, with Cl^−^ and K^+^ apart, as the more likely structure in solution, consistent with ^1^H NMR data.

**Fig. 11:**
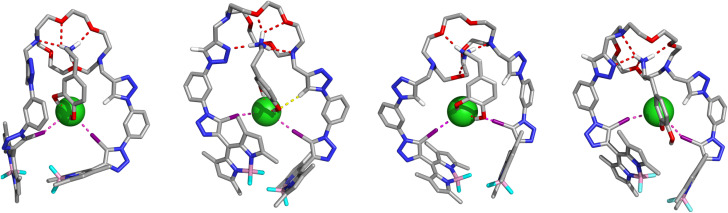
DFT gas-phase optimised structures of 3 dopamine·HCl in binding conformations D_α_, D_β_, D_γ_, and D_δ_ (left to right).

The binding conformations of the heteroditopic host 3 for the recognition of the dopamine·HCl ion-pair were studied using a computational modelling approach analogous to that used in Cl^−^ recognition by 3·K^+^ (see SI). In agreement with the ^1^H NMR data, three alternative binding arrangements, D_α_–D_γ_, were selected from gas-phase MD simulations, with both catechol hydroxyl groups of the dopamine·HCl motif establishing two putative hydrogen bonds with the halogen bonded Cl^−^, while the alkylammonium's NH_3_^+^ group is recognised by the crown ether moiety of 3, as illustrated in Fig. 11, with the M06-2X/def2-TZVP(D) optimised structures. In D_α_ and D_β_, dopamine·HCl is positioned between the XB-BODIPY pendant arms, with its phenyl ring nearly parallel to a phenyl group of 3, while in D_γ_, the alkylammonium's phenyl ring lies nearly coplanar with the plane defined by the host's triazole rings. Moreover, regardless of the binding disposition, 3 adopts unfolded conformations with comparable shapes, mainly differing in the spatial dispositions of two proto-triazole units, one of which, in D_β_, points to a catechol hydroxyl group at 2.31 Å. In D_γ_, both proto-triazoles point towards the interior of 3, but remain distant from Cl^−^.

Each of the D_α_–D_γ_ structures was immersed in periodic boxes of acetone or acetonitrile/methanol 1 : 1 (v/v), and across all six 50 ns MD runs (three per solvent), the host–guest complex preserved the XB interactions with the anion. Host 3 exhibits a large flexibility, leading D_γ_ to lose its initial convergent disposition of both C_trz_–H groups during the equilibration stage.

Regardless of the initial dopamine·HCl pose, one O–H⋯Cl^−^ bond consistently breaks during the simulation, being replaced by an intramolecular O–H⋯O–H hydrogen bond between the hydroxyl groups. This new binding arrangement, D_δ_, is illustrated with the gas-phase DFT optimised geometry in Fig. 11, sourced from the MD runs in acetone. The four alternative binding arrangements exhibit XB interactions with I⋯Cl^−^ distances ranging between 3.14 and 3.56 Å (Table S7), comparable to those calculated for 3·KCl. The geometry of the XB interactions remains nearly linear, particularly in D_γ_, where both proto-triazoles point away from Cl^−^. Furthermore, the dimensions of the O–H⋯Cl^−^ hydrogen bonds fall within the expected range (Table S7). NBO analysis (Table S7) revealed that the O–H⋯Cl^−^ contacts (*n*_Cl^−^_→σ*_O–H_), ranging from 18.1 (D_δ_) to 54.3 kcal mol^−1^ (D_β_), significantly assist halogen bond recognition of the dopamine·HCl ion pair, complementing the XB contributions ranging from 13.1 (D_δ_) to 17.7 kcal mol^−1^ (D_β_).

The impact of the solvent on the four alternative binding arrangements was also DFT evaluated, but only in acetone (PCM solvent model), due to the size of host–guest complexes between the dopamine·HCl ion-pair and the XB-based host 3. As found for the 3·KCl complex, the alternative structures computed in the gas phase and in acetone are almost identical, with RMSD values ranging from 0.16 to 0.67 Å for D_α_–D_δ_. Moreover, differences in the XB distances do not exceed 0.17 Å across four binding scenarios (Table S8). The Δ*E*_conf_ values for D_α_–D_γ_ show that D_β_ is favoured over D_α_ (2.9 kcal mol^−1^) and D_γ_ (9.4 kcal mol^−1^), while the D_δ_, extracted from the MD simulations, is slightly preferred over D_β_ by 1.0 kcal mol^−1^ (Table S9).

In summary, MD and DFT results show that XB-chloride assisted recognition of dopamine·HCl by 3 involves one or two O–H⋯Cl^−^ hydrogen bonds with the catechol moiety, which is entirely consistent with ^1^H NMR findings.

## Conclusions

In conclusion, a novel halogen bonding heteroditopic receptor 3, consisting of a diaza-crown ether functionalised with BODIPY fluorophore-appended anion binding sites, was designed and synthesised. The receptor proved capable of the optical sensing of cations, anions and ion-pairs *via* a turn-on fluorescence emission response in competitive 1 : 1 (v/v) acetonitrile : methanol media.

3 exhibited selective fluorescence sensing of K^+^ over Na^+^, attributed to a distinct triazole N⋯K^+^ binding mode, supported by ^1^H NMR, DFT and molecular dynamics studies. The sensor displayed a high degree of co-operativity for KCl binding, attributed to cation-induced polarisation of the triazole anion-binding site, combined with proximal electrostatic interactions.

Importantly, 3 demonstrated selective binding of the neurotransmitter salt dopamine·HCl over related phenethylamine derivatives, with a mixed halogen- and hydrogen-bonding anion binding site forming convergently between the receptor and catechol motif of the co-bound dopamine cation.

This approach showcases the ability of judiciously designed intercomponent interactions between a co-bound ion-pair and a heteroditopic receptor's multiple proximal convergent binding motifs as a potent strategy for achieving selectivity in the sensing of biologically relevant ion-pair substrates. Further work to develop sensors capable of sensing in biologically-relevant media is ongoing in our laboratories.

## Author contributions

J. T. W. and A. J. T. performed the synthesis, characterisation and ^1^H NMR and optical binding studies in the manuscript. I. M. and V. F. performed the computational studies. All four authors were involved in the drafting and revision of the manuscript. P. D. B. conceived and supervised the project, and revised the draft manuscript.

## Conflicts of interest

There are no conflicts to declare.

## Supplementary Material

SC-016-D5SC05033B-s001

SC-016-D5SC05033B-s002

## Data Availability

The data supporting this article, including the XYZ coordinates and electronic energies of the DFT-optimised structures, have been included as part of the SI. Supplementary information is available. See DOI: https://doi.org/10.1039/d5sc05033b.
