# Supplementary material for: A halogen bonding BODIPY-appended aza-crown ether for selective optical sensing of inorganic and organic ion-pair species
Source: Chem Sci. 2025 Sep 8;16(41):19271–9. doi: 10.1039/d5sc05033b (PMC12447292; doi:10.1039/d5sc05033b)
Supplement: SC-016-D5SC05033B-s002 [file SC-016-D5SC05033B-s002.pdf]

Supplementary Information (SI) for Chemical Science.
This journal is © The Royal Society of Chemistry 2025

Supporting Information

A Halogen Bonding BODIPY-appended Aza-Crown Ether for Selective Optical Sensing of Inorganic and Organic Ion-Pair Species

Jamie T. Wilmore[†,a], Andrew J. Taylor[†,a], Igor Marques[b], Vítor Félix[b], and Paul D. Beer*[,a]

[a]Department of Chemistry, Chemistry Research Laboratory, University of Oxford, Mansfield Road, Oxford, OX1 3TA, U.K.

[b] Department of Chemistry, CICECO – Aveiro Institute of Materials, University of Aveiro, 3810-193, Aveiro, Portugal

†equal contribution

*paul.beer@chem.ox.ac.uk

Contents

1. Instrumentation and General Experimental Details 3

General Information 3

2. Synthesis and Characterisation of Compounds 4

Compound 1 4

Compound 3 4

3. Optical Characterisation of 3 8

4. Fluorescence Titration Studies 9

5. ^{1}H NMR Titration Study 12

6. Molecular Modelling: Additional Data & Methods 14

Computational Analysis of K^+ Binding Modes in 3 14

Energy Calculations 15

Additional Figures 16

Additional Tables 19

Computational Methods/Molecular Modelling details 21

General 21

Classical force field calculations 21

Boron force field parameters 22

RESP atomic charges and extra point of charge derivatisation 24

Conformational Analyses 25

Quantum Calculations on K^+ and ion-pair complexes of **3** 26

Molecular dynamics simulations 26

Notes and References 28

1. Instrumentation and General Experimental Details

General Information

Solvents and reagents were purchased from commercial suppliers and used as received. Dry solvents were obtained by purging with nitrogen and passing through a MBraun MPSP-800 column. H_2O was de-ionised and micro-filtered using a Milli-Q® Millipore machine.

Experiments were conducted at room temperature unless otherwise stated. Merck silica gel 60 or Sigma Aldrich alumina gel (neutral, Brockmann activity I) were used for flash column chromatography. TBA salts were stored in vacuum desiccators prior to use. NMR spectra were either recorded on a Bruker Avance III HD Nanobay NMR spectrometer equipped with a 9.4 T magnet or a Bruker NEO 600 with broadband helium cryoprobe. ^{1}H NMR titrations were recorded on a Bruker Avance III NMR equipped with a 11.75 T magnet.

Chemical shifts are quoted in parts per million relative to the residual solvent peak.

UV-vis and fluorescence measurements were carried out on a Duetta (Horiba) using quartz cuvettes with a path length of 10 mm. Unless otherwise noted, all fluorescence spectra were acquired with a wavelength of excitation of 485 nm, 5 nm excitation and emission slits and were recorded in, at least, triplicate repeat measurements to ensure signal stability. Titration studies were carried out by titrating a 1 µM solution of the receptor with aliquots of a concentrated solution of the titrant in the same receptor solution to ensure a constant receptor concentration.

Spectrophotometric grade solvents were used for all optical studies.

Tris[(1-benzyl-1H-1,2,3-triazol-4-yl)methyl]amine is abbreviated as TBTA.

Diaza-18-crown-6 (Kryptofix® 22) was purchased from Sigma Aldrich.

All data analysis and fitting were carried out using *OriginPro* 2023.[1]

2. Synthesis and Characterisation of Compounds

Compound 1

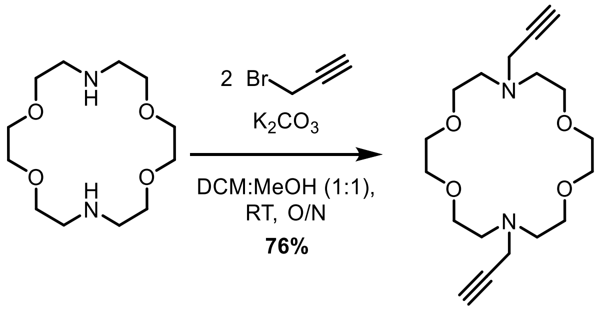

1

Scheme S1. Synthesis of **1.**

Diaza-18-crown-6 (Kryptofix® 22) (500 mg, 1.9 mmol) was dissolved in 20 mL DCM:MeOH 1:1 (*v/v*). Propargyl bromide (80 wt % in toluene) (0.5 mL, 5.1 mmol, 2.5 eq) and K_2CO_3 (1.3 g, 5 eq) were added and the mixture was left to stir overnight at room temperature. The mixture was filtered and the solvent removed *in vacuo*. The residue was redissolved in DCM (20 mL) and washed with H_2O (3 x 10 mL). The crude was purified by alumina column chromatography (DCM:MeOH 99:1) to give compound **1** as a colourless wax in 76% yield.

^{1}H and ^{13}C NMR characterisation were consistent with previous reports.[2, 3]

Compound 3

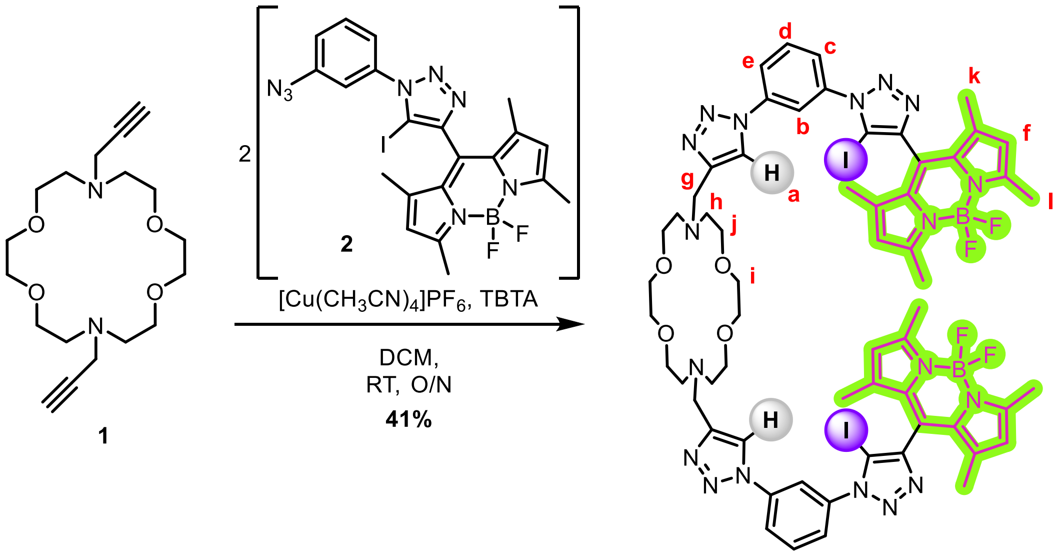

3

Scheme S2. Synthesis of **3**.

The iodo-triazole BODIPY-appended azide, **2**, was prepared according to a literature procedure.[4] Compound **1** (17 mg, 0.05 mmol) was dissolved in dry DCM (6 mL) and compound **2** (60 mg, 0.11 mmol, 2.1 eq), $[Cu(CH_3CN)_4]PF_6$ (4 mg, 0.01 mmol, 0.2 eq) and TBTA (6 mg, 0.01 mmol, 0.2 eq) were added. The mixture was left to stir under N_2 overnight at room temperature in the dark. The reaction mixture was diluted with DCM (15 mL) and washed with NH_4OH/EDTA (aq.) (2 x 20 mL) and H_2O (20 mL). The combined organic layers were dried over $MgSO_4$ and the volatiles removed *in vacuo*. The crude residue was purified by alumina column chromatography (DCM:MeOH 98:2). Following purification by chromatography, the solvent was removed *in vacuo* and the solid redissolved in DCM (20 mL), which was washed with water (3 x 10mL) to remove any residual salts from the alumina. The product, **3**, was obtained as a pink solid in 41% yield.

^{1}H NMR: (500 MHz, acetone-d_6) δ: 8.68 (s, 2H_a), 8.30 (s, 2H_b), 8.23 (d, *J* = 8.1 Hz, 2H_c), 7.91 (t, *J* = 8.1 Hz, 2H_d), 7.79 (d, *J* = 8.0 Hz, 2H_e), 6.21 (s, 4H_f), 3.91 (s, 4H_g), 3.63 (t, *J* = 5.6 Hz, 8H_h), 3.60 (s, 8H_i), 2.78 (t, *J* = 5.6 Hz, 8H_j), 2.54 (s, 12H_k), 1.62 (s, 12H_l) ppm.

^{13}C{^{1}H} NMR: (126 MHz, acetone-d_6) δ: 158.2, 147.3, 143.7, 139.3, 138.8, 133.1, 132.2, 129.4, 126.9, 122.8, 122.6, 118.7, 71.4, 70.7, 55.2, 51.1, 14.9, 14.3 ppm. (3 aromatic peaks are believed to be coincident).

HRMS: m/z calculated for $[C_{60}H_{66}B_2F_4I_2N_{18}O_4Na]^+$, $[M+Na]^+$: 1477.3624, found: 1477.3620.

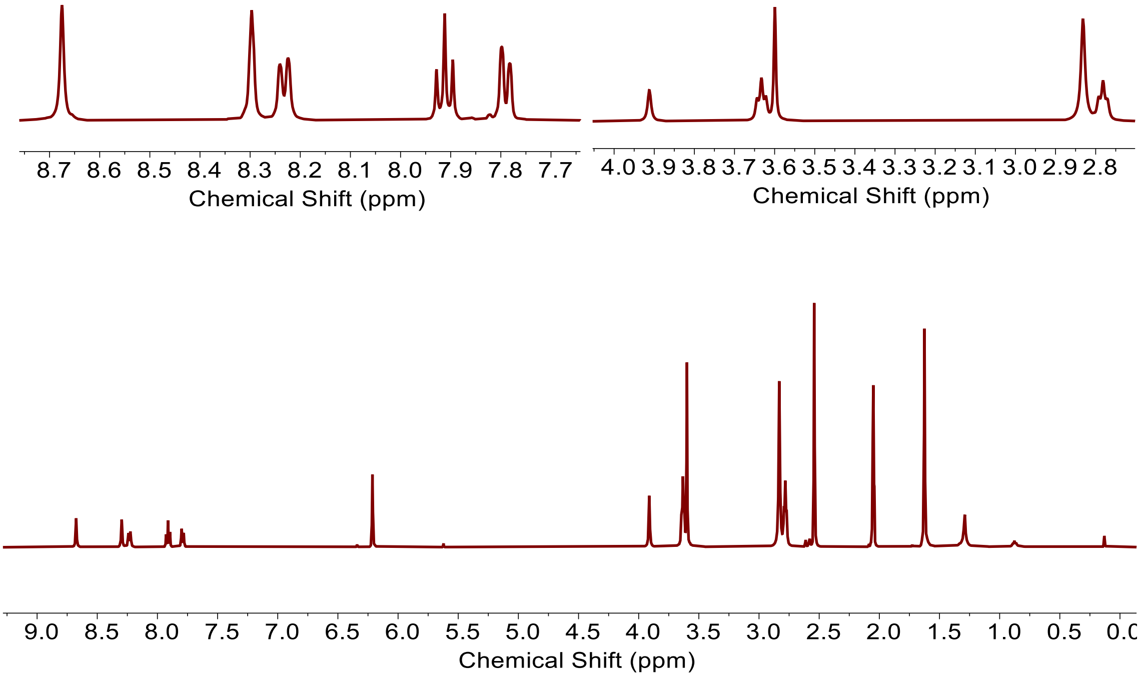

Figure S1. ^{1}H (500 MHz, 298 K, acetone-d_6) NMR spectrum of **3**.

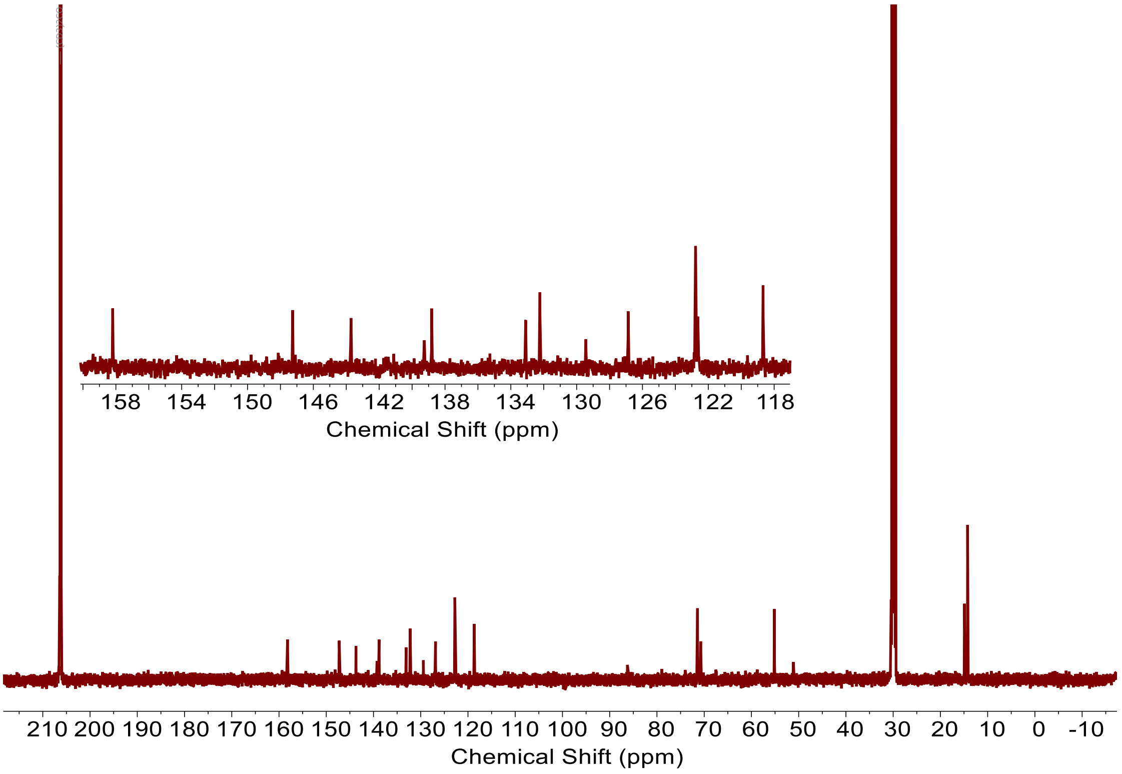

Figure S2. $^{13}C\{^{1}H\}$ (126 MHz, 298 K, acetone-d_6) NMR spectrum of **3**.

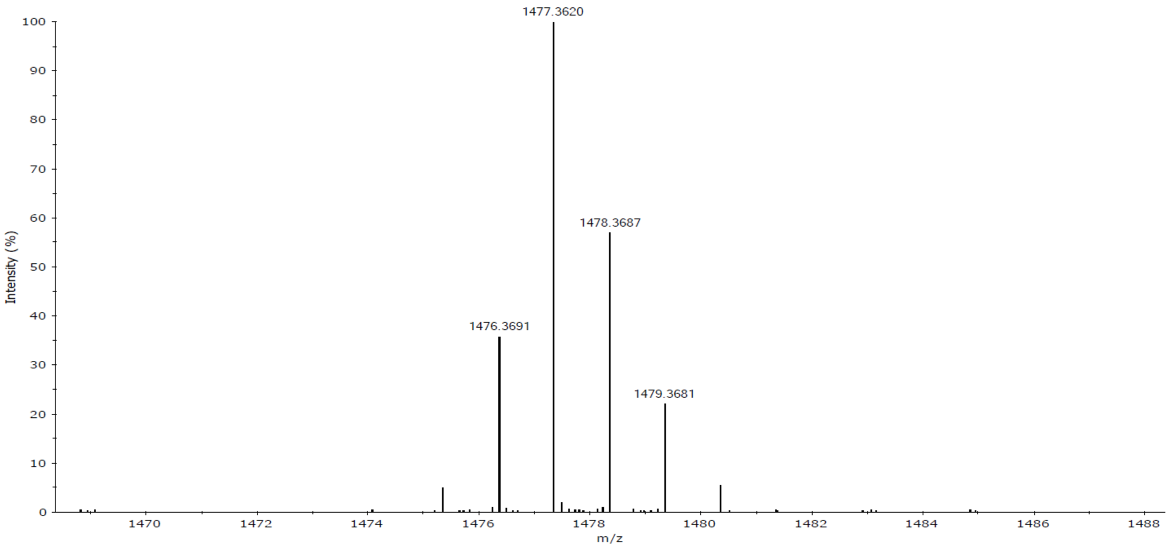

Figure S3. ESI-HRMS spectrum of **3**.

3. Optical Characterisation of 3

The absorption and emission spectra for compound **3** is shown below. It showed the expected absorption and emission features for BODIPY-containing compounds, with an absorption maximum of 511 nm and an emission maximum of 523 nm. This corresponds to a Stokes shift of 12 nm, which is in line with previous reports.

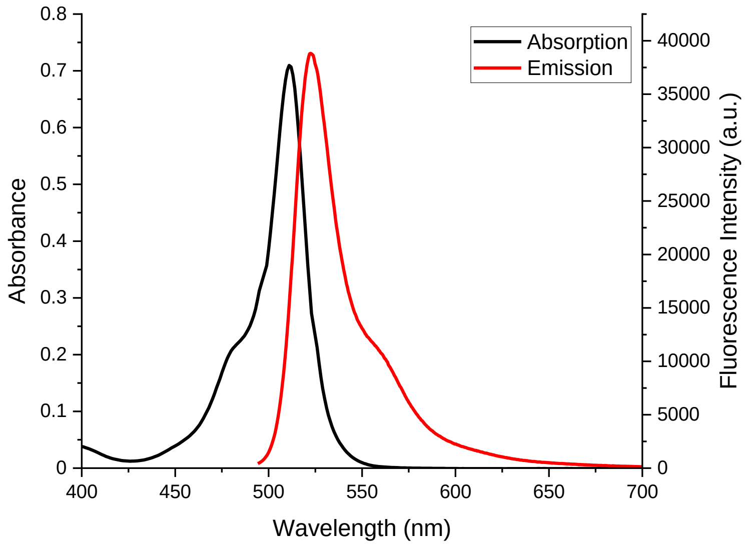

Figure S4. Absorption and emission spectra of **3**. (10 µM, acetonitrile, 298K)

4. Fluorescence Titration Studies

As described in the main text, fluorescence titration studies of sensor **3** were conducted in an acetonitrile/methanol 1:1 (*v/v*) solvent mixture with a variety of ion-pairs, containing both non-coordinating and coordinating cations. In each case, the ion-pair was dissolved in the same solvent mixture and added directly to a solution of the receptor. Representative, stacked emission spectra, with arrows showing the direction of change upon addition of analyte (if any), are shown below.

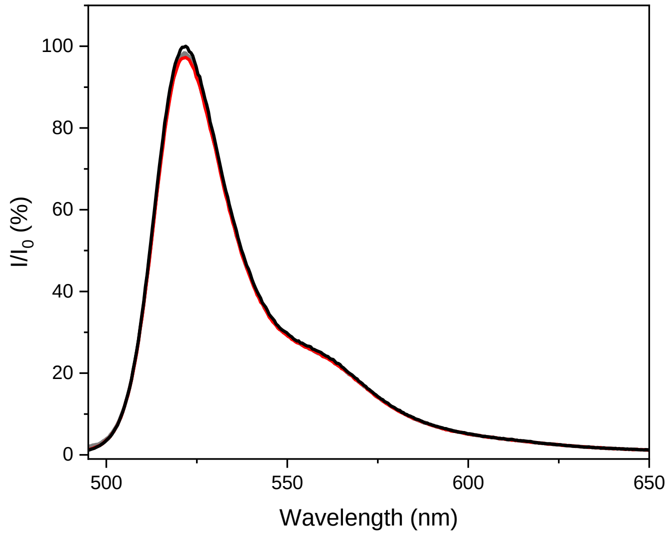

Figure S5. Stacked emission spectra of **3** with increasing concentrations of $NaBAr^F{}_4$ (up to a maximum of 2.86 mM). (1 µM, acetonitrile/methanol 1:1 (*v/v*), 298 K)

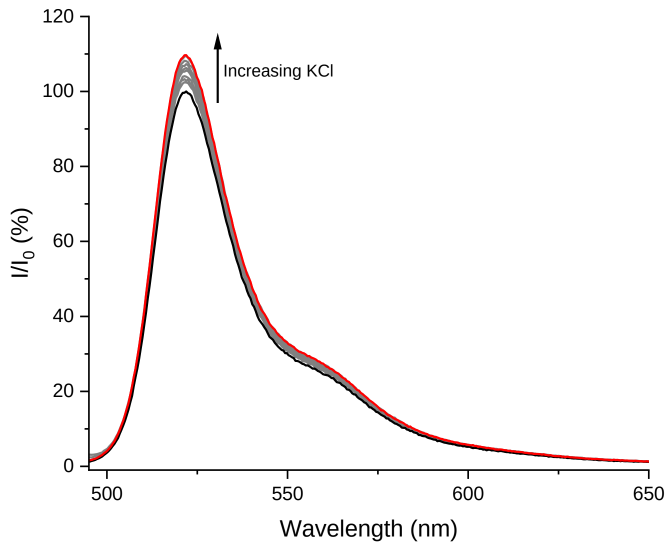

Figure S6. Stacked emission spectra of **3** with increasing concentrations of KCl (up to a maximum of 7.14 mM). (1 µM, acetonitrile/methanol 1:1 (*v/v*), 298 K)

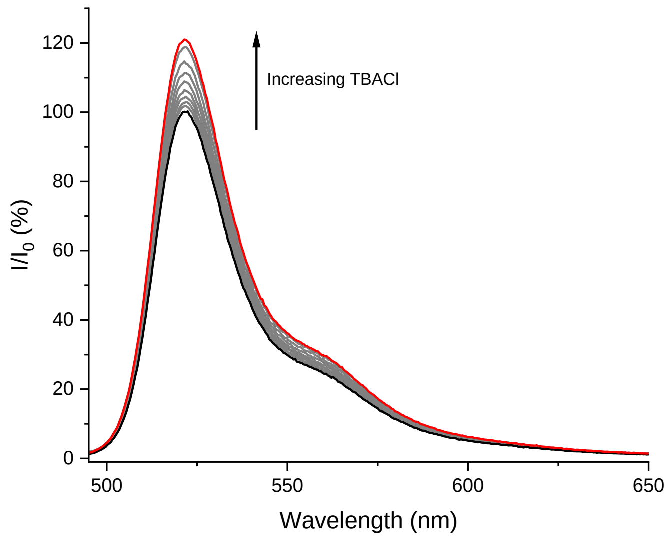

Figure S7. Stacked emission spectra of **3** with increasing concentrations of TBACl (up to a maximum of 28.6 mM). (1 µM, acetonitrile/methanol 1:1 (*v/v*), 298 K)

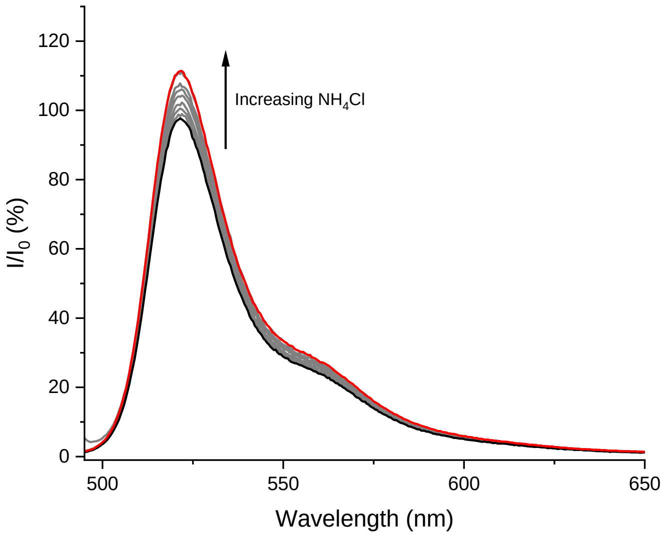

Figure S8. Stacked emission spectra of **3** with increasing concentrations of NH_4Cl (up to a maximum of 7.14 mM). (1 µM, acetonitrile/methanol 1:1 (*v/v*), 298 K)

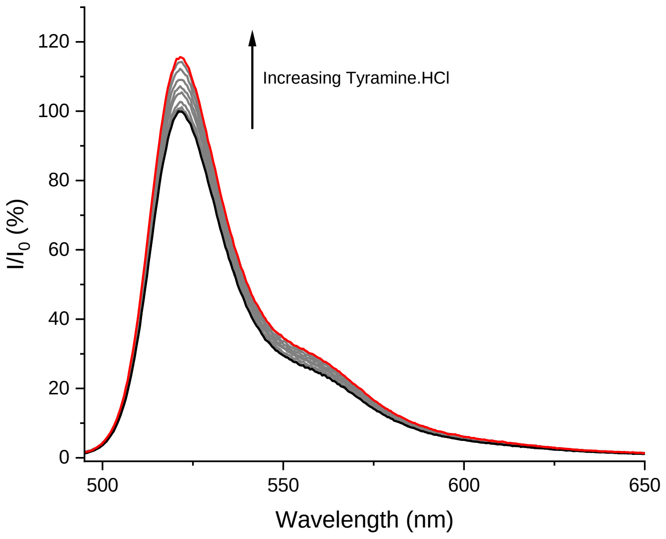

Figure S9. Stacked emission spectra of **3** with increasing concentrations of the hydrochloride salt of tyramine (up to a maximum of 1.43 mM). (1 µM, acetonitrile/methanol 1:1 (*v/v*), 298 K)

5. ^{1}H NMR Titration Study

In order to elucidate the origin of the differing fluorescence responses upon Na^+ and K^+ binding, ^{1}H NMR titrations were undertaken with compound **3** in acetone-*d₆*. Aliquots of Na^+ and K^+ as their $BAr^F{}_4{}^-$ salts were added to a 1 mM solution of compound **3** and the ^{1}H NMR spectrum recorded. Stacked ^{1}H NMR spectra are shown below. Of particular note is the differing behaviour of the triazole proton H_a upon alkali metal cation binding (Figures S10-11). Upon K^+ binding, the H_a resonance shifts significantly downfield, indicating de-shielding, which is presumably caused by coordination of the triazole N-atom to the larger K^+ cation. In contrast, a far smaller shift is seen when Na^+ is added, suggesting that the triazole does not coordinate to this smaller cation. Strong binding was observed for both titrations, with minimal changes in the spectra observed after 1 equivalent of the alkali metal cation had been added.

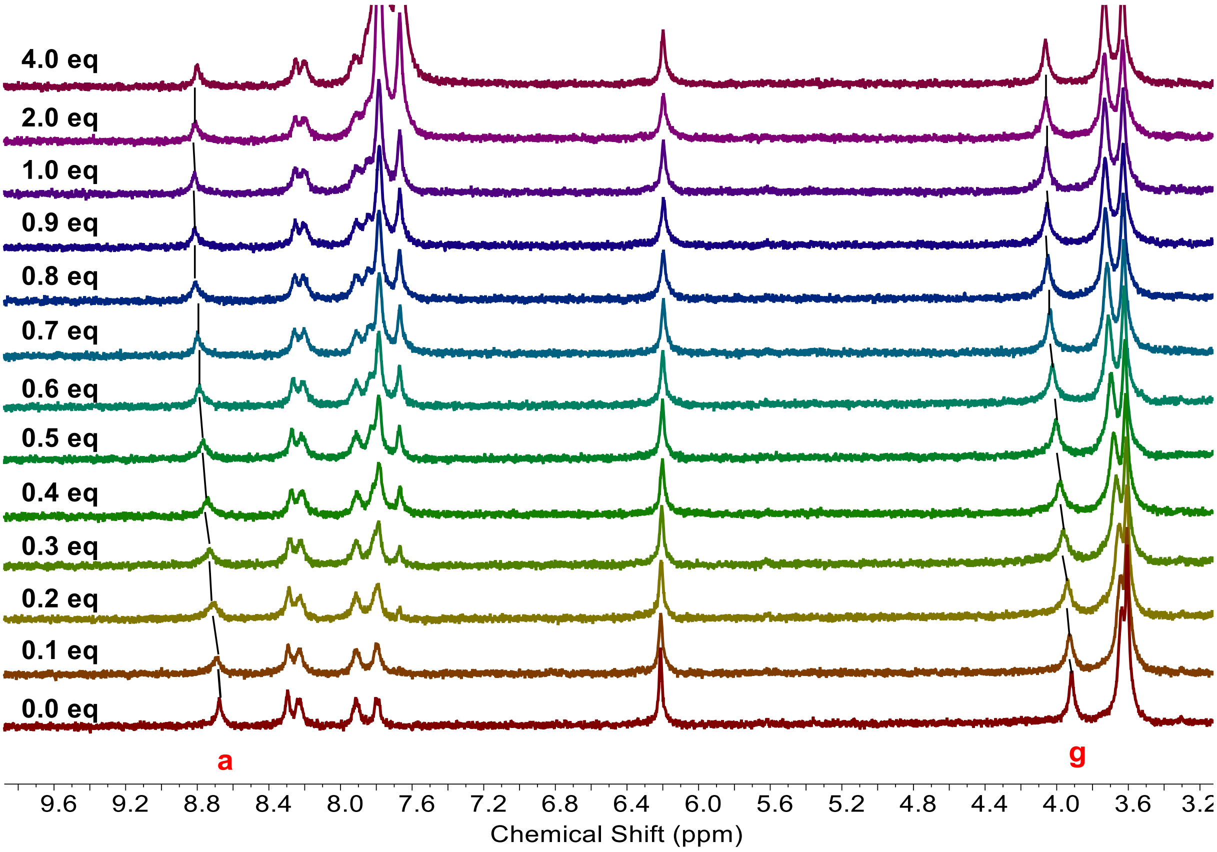

Figure S10. Stacked ^{1}H NMR spectra of **3** with increasing concentrations of $KBAr^F{}_4$. (1 mM, 500 MHz, acetone-d_6, 298 K)

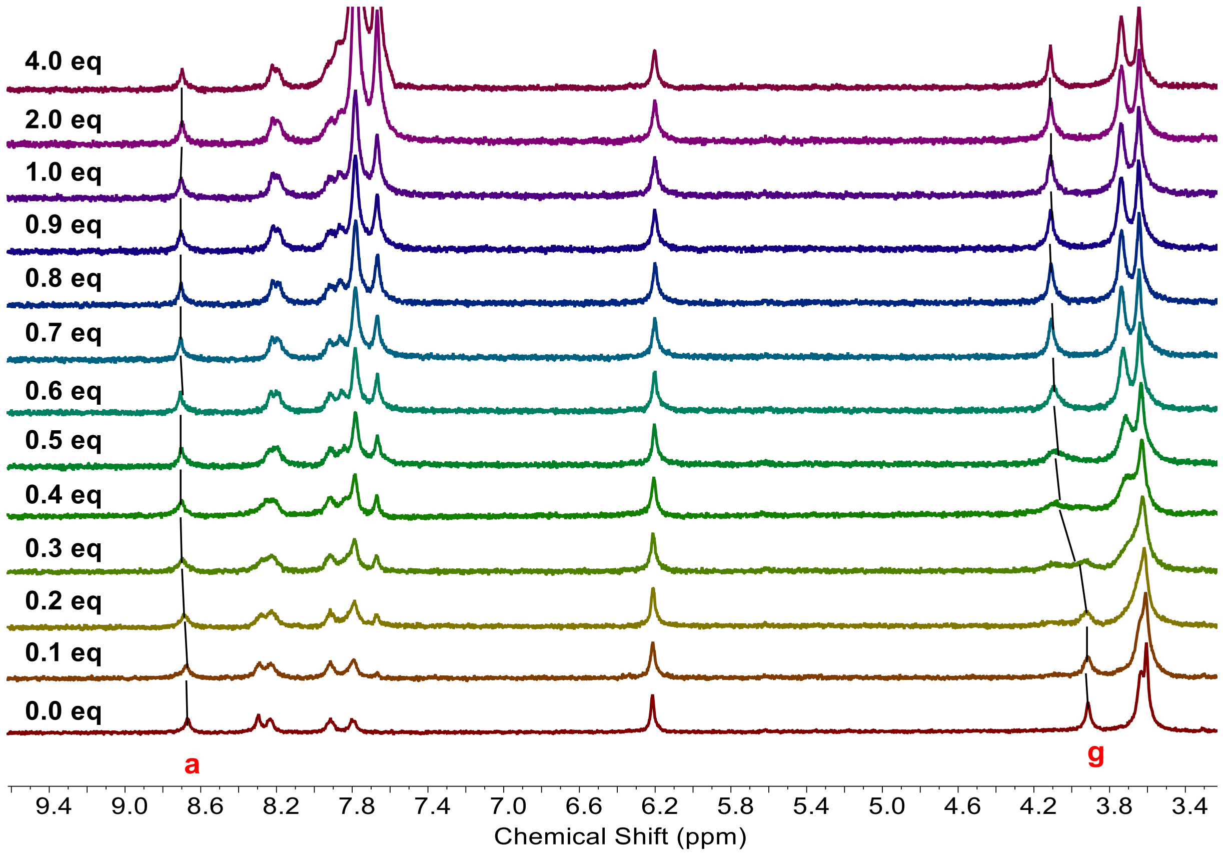

Figure S11. Stacked ^{1}H NMR spectra of **3** with increasing concentrations of $NaBAr^F_4$. (1 mM, 500 MHz, acetone-d_6, 298K)

6. Molecular Modelling: Additional Data & Methods

Computational Analysis of K^+ Binding Modes in 3

Structural insights into the potassium cation binding modes were obtained from molecular dynamics (MD) simulations followed by density functional theory (DFT) calculations. The host-guest complex of **3** with K^+ inserted in the aza-crown-ether cavity was simulated under periodic boundary conditions in both solvent media (acetone or acetonitrile/methanol 1:1 (*v*/*v*) mixture) in presence of $BAr^F{}_4{}^-$. Three independent MD runs of 100 ns were carried out with AMBER20 (see below).[5] Regardless of the solvent media, **3** coordinated to K^+ exhibits high conformational flexibility, with the two XB BODIPY macrocyclic pendant arms adopting random spatial dispositions during the MD runs. In addition, the triazole rings appended to the macrocyclic moiety by the methylene bridges establish zero, one, or two coordinating contacts with the encapsulated K^+ cation, as shown in Figure S12, where the distances of the alkali cation to the triazoles' nitrogen atoms closest to the methylene bridges ($K^+\cdots N_{trz}$) are plotted along the simulation time. These three alternative coordination modes, designated K_α, K_β, and K_γ, are illustrated in Figure S13, with representative snapshots taken from MD simulations performed in acetone or in the solvent mixture.

The interactions between the alkali K^+ cation and heteroditopic sensor **3** were further investigated by DFT calculations using the alternative coordination modes found in the MD simulations. These K_α–K_γ binding arrangements were characterised in the gas-phase at the M06-2X/def2-TZVP(D) theory level with Gaussian 16 (see below).[6] Moreover, given the conformational flexibility of the host's arms observed in the MD simulations, the DFT calculations were performed using a model complex of K^+ with a truncated model of **3** with the XB-BODIPY-appended arms replaced by methyl groups (**3'**). The optimised geometries of the K_α–K_γ binding arrangements are shown in Figure S14, with K_γ being favoured over K_α and K_β by ΔE_{conf} values of 12.6 and 4.2 kcal mol^{-1}, respectively (Table S1). However, when the solvent effects were considered in the DFT geometry optimizations (PCM solvent model), the binding mode preference for K_γ is maintained, albeit the ΔE_{conf} values to the K_α and K_β binding modes respectively decrease to ca. 3.8 and 1.4 kcal mol^{-1} in methanol and acetonitrile, and to 4.0 and 1.2 kcal mol^{-1} in acetone (Table S1).

The distances computed in the gas-phase between K^+ and neighbouring N and O donors of **3'** are listed in Table S2. In the K_α–K_γ binding modes, the $K^+\cdots O$ distances range between 2.68 Å (K_α) and 2.83 Å (K_γ), while the aza-crown $K^+\cdots N$ distances range from 2.82 (K_α) to 3.00 Å (K_β). Moreover, K_β displays a $K^+\cdots N_{trz}$ distance of 2.76 Å, while K_γ presents two $K^+\cdots N_{trz}$ distances of 2.84 Å, being slightly shorter than the corresponding $K^+\cdots N$ coordination distances computed in the aza-crown moiety.

The $K^+\cdots N_{trz}$ bonding contacts were characterised by Natural Bond Orbital (NBO) analysis, by estimating the Second-order Perturbation Theory energies (E^2) resulting from the charge transfer interactions between the lone pair orbitals of N_{trz} and the lone vacancy orbitals of K^+ ($n_{Ntrz} \rightarrow n^*_{K+}$, Table S2). In K_β, the single $K^+\cdots N_{trz}$ interaction presents an E^2 of 5.3 kcal mol^{-1}, while in K_γ, both $K^+\cdots N_{trz}$ interactions display E^2 values of 6.1 kcal mol^{-1}. The additional interaction in K_γ further stabilizes the binding arrangement, in line with the theoretical binding preference and with the ^{1}H NMR structural evidence.

Energy Calculations

The conformational energy differences between alternative binding arrangements, mentioned in the main text, were estimated from the thermal-corrected electronic energies of the ground state optimised geometries, according to the equation:

$$\Delta E_{conf} = E_{conf1} - E_{conf2} \qquad \text{(Eq. S1)}$$

where E_{conf1} and E_{conf2} represent the total thermal corrected electronic energies of the respective conformational binding scenarios. These values were computed as the sum of the electronic energy (ε_0), the zero-point vibrational energy (ZPE), and thermal corrections accounting for translational, rotational, vibrational, and electronic motions.

Additional Figures

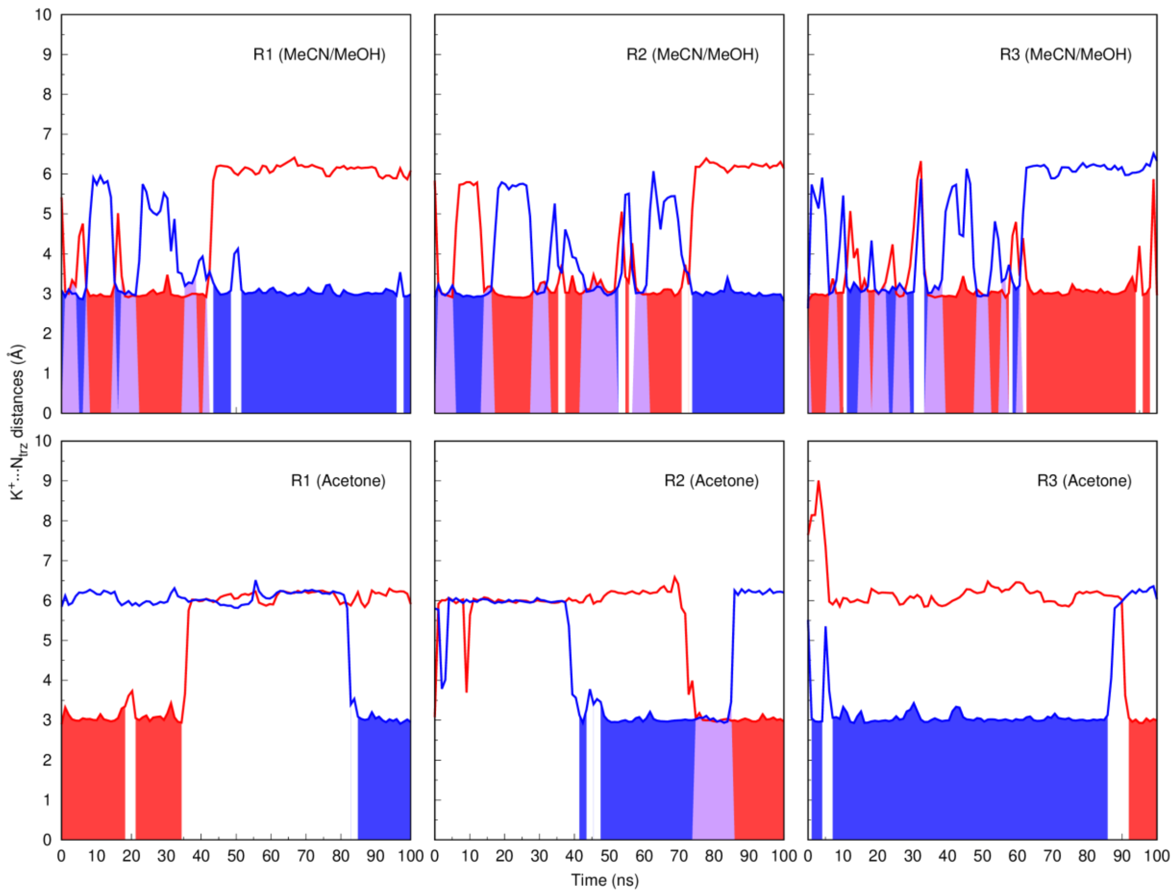

Figure S12. Evolution of the $K^+\cdots N_{trz}1$ (red line) and $K^+\cdots N_{trz}2$ (blue line) distances through time in 100 ns MD simulations of **3**·K^+ in three independent MD runs in the acetonitrile/methanol 1:1 (*v*/*v*) solvent mixture (top) and in acetone (bottom). The red and blue shaded areas correspond to one $K^+\cdots N_{trz}$ coordinating contact, while the purple shaded areas correspond to two $K^+\cdots N_{trz}$ coordinating contacts.

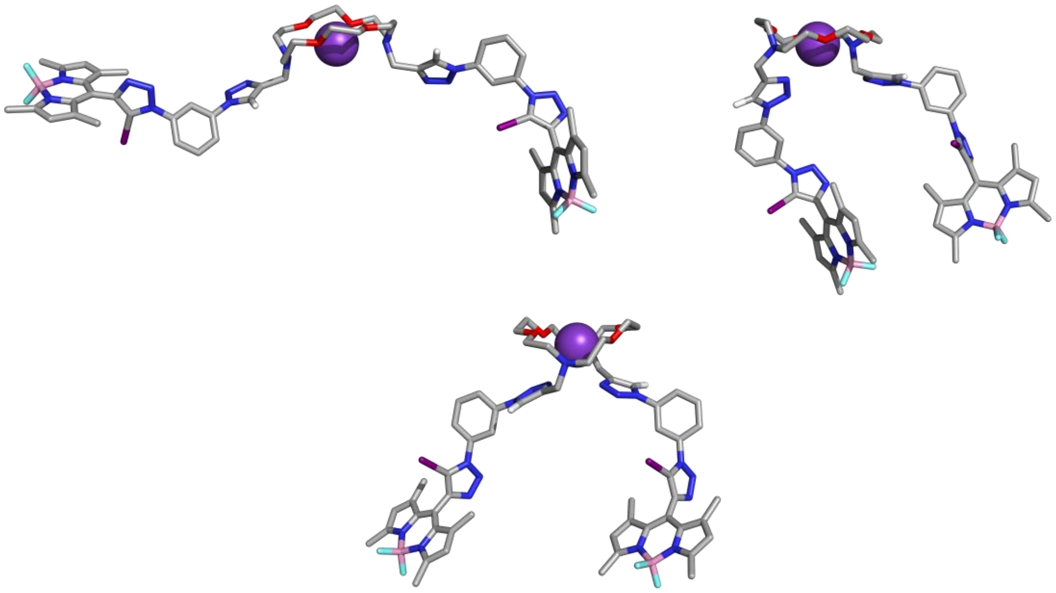

Figure S13. Representative conformations of **3**·K$^+$ observed in the MD simulations in solution: K$_\alpha$ (top left), K$_\beta$ (top right), and K$_\gamma$ (bottom).

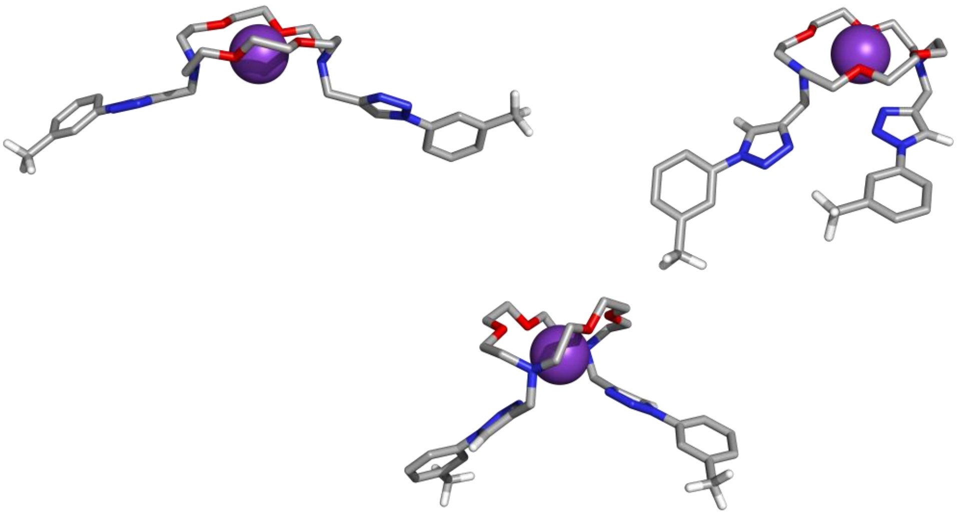

Figure S14. DFT optimized structures of **3'**·K$^+$: K$_\alpha$ (top left), K$_\beta$ (top right), and K$_\gamma$ (bottom).

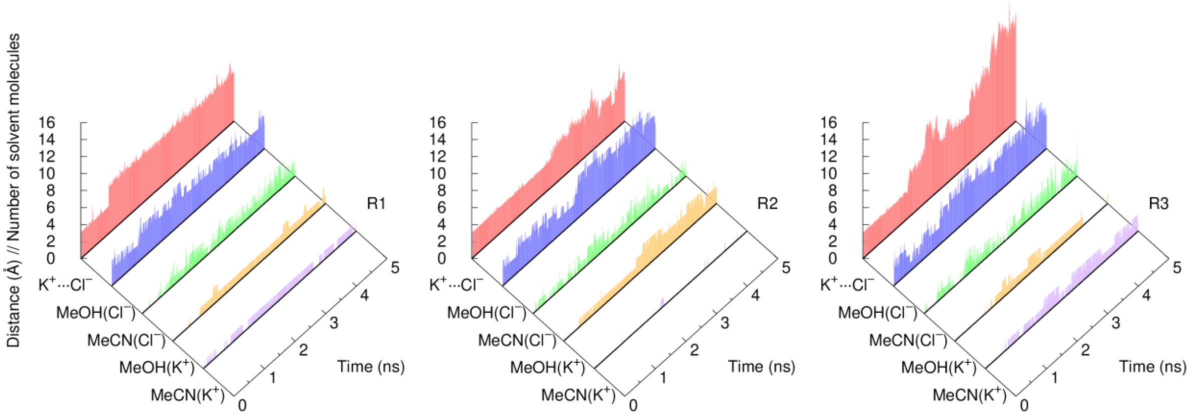

Figure S15. Evolution of the $K^+\cdots Cl^-$ distance (red) during the first 5 ns of the 50 ns MD simulations of **3**·KCl in three independent MD runs in the acetonitrile/methanol 1:1 (*v/v*) solvent mixture, together with the solvation of the Cl^- and K^+ ions by methanol and acetonitrile molecules (blue and green for Cl^-; orange and purple for K^+) at a radius of 3.5 Å.

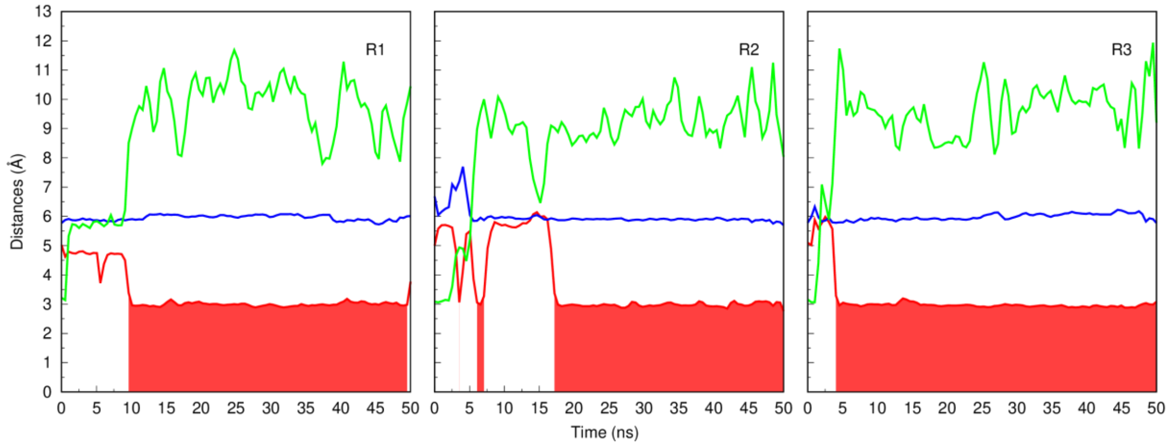

Figure S16. Evolution of the $K^+\cdots Cl^-$ distance (green) throughout the 50 ns MD simulations of **3**·KCl in three independent MD runs in the acetonitrile/methanol 1:1 (*v/v*) solvent mixture, together with $K^+\cdots N_{trz}1$ (red line) and $K^+\cdots N_{trz}2$ (blue line) distances. The red shaded areas correspond to one $K^+\cdots N_{trz}$ coordinating contact.

Additional Tables

Table S1. Uncorrected electronic energy differences ($\Delta\varepsilon_0$),[a] zero-point corrections (ΔZPE),[b] and thermal corrected energy differences (ΔE)[c] (all in kcal mol^{-1}) for selected alternative arrangements of **3'**·K$^+$.

Condition	Gas phase		MeOH		MeCN	
Energy term	K_γ-K_α	K_γ-K_β	K_γ-K_α	K_γ-K_β	K_γ-K_α	K_γ-K_β
$\Delta\varepsilon_0$	-12.3	-3.9	-3.5	-1.1	-3.5	-1.1
ΔZPE	-0.2	-0.4	-0.1	-0.4	-0.1	-0.4
ΔE	-12.6	-4.2	-3.8	-1.4	-3.7	-1.4

a) Energy values were not corrected for basis set superposition errors; b) ΔZPE is included in the ΔE term; c) $\Delta E = \Delta\varepsilon_0 + \Delta E_{Tot}$, where ΔE_{Tot} accounts for internal energy differences due to translational, rotational, vibrational, and electronic motions. Absolute energy terms are defined above.

Table S2. Distances (Å) and second-order perturbation interaction energies (E^2, kcal mol^{-1}) for the interactions between K$^+$ and the nitrogen and oxygen atoms of the aza-crown in gas-phase DFT-optimised **3'**·K$^+$ complexes. Data are shown for three coordination modes (K_α, K_β, and K_γ), including the interactions with the N_{trz} atoms.

Coordination mode	K_α		K_β		K_γ	
Interaction	Distance	E^2	Distance	E^2	Distance	E^2
K$^+$···N1	2.85	1.3	3.00	1.6	2.95	2.0
K$^+$···N2	2.85	1.2	2.82	1.6	2.95	2.0
K$^+$···O1	2.68	2.9	2.69	3.5	2.83	3.7
K$^+$···O2	2.72	2.8	2.75	3.4	2.75	3.7
K$^+$···O3	2.71	2.9	2.74	3.5	2.83	3.7
K$^+$···O4	2.75	2.7	2.73	3.2	2.75	3.7
K$^+$···N_{trz}1	5.78	–	5.42	0.1	2.84	6.1
K$^+$···N_{trz}2	5.70	–	2.76	5.3	2.84	6.1

Table S3. Distances (Å) and angles (°) for the interactions between K$^+$ and the N_{trz} atoms, for the halogen and hydrogen bonding interactions with Cl$^-$, as well as for K$^+$···Cl$^-$, in the gas-phase DFT-optimised **3**·KCl complexes. Data are shown for three coordination modes: KCl_α, KCl_β, and KCl_γ.

Coordination mode	KCl_α		KCl_β		KCl_γ	
Interaction	Distance	Angle	Distance	Angle	Distance	Angle
K$^+$···N_{trz}1	4.93	–	6.05	–	2.78	–
K$^+$···N_{trz}2	5.76	–	6.11	–	5.70	–
C_{trz}-I1···Cl$^-$	3.12	165.3	2.73	154.5	3.00	178.1
C_{trz}-I2···Cl$^-$	3.08	173.3	3.09	167.8	3.32	157.2
C_{trz}-H1···Cl$^-$	2.72	137.2	2.73	154.2	8.30	21.9
C_{trz}-H2···Cl$^-$	3.47	165.1	3.37	139.4	2.55	142.7
K$^+$···Cl$^-$	2.98	–	2.93	–	5.74	–

Table S4. Uncorrected electronic energy differences ($\Delta\varepsilon_0$),[a] zero-point corrections (ΔZPE),[b] and thermal corrected energy differences (ΔE)[c] (all in kcal mol^{-1}) for selected alternative arrangements of **3**·KCl.

Condition	Gas phase		MeOH	MeCN
Energy term	KCl_α-KCl_β	KCl_α-KCl_γ	KCl_α-KCl_γ	KCl_α-KCl_γ
$\Delta\varepsilon_0$	-5.5	-24.7	-6.6	-6.5
ΔZPE	0.5	0.9	0.9	0.9
ΔE	-5.6	-24.7	-6.6	-6.5

a) Energy values were not corrected for basis set superposition errors; b) ΔZPE is included in the ΔE term; c) $\Delta E = \Delta\varepsilon_0 + \Delta E_{Tot}$, where ΔE_{Tot} accounts for internal energy differences due to translational, rotational, vibrational, and electronic motions. Absolute energy terms are defined above.

Table S5. Second-order perturbation interaction energies (E^2, kcal mol^{-1}) for the interactions between K^+ and the N_{trz} atoms, for the halogen and hydrogen bonding interactions with Cl^-, as well as for $K^+\cdots Cl^-$, in the gas-phase DFT-optimised **3**·KCl complexes. Data are shown for two coordination modes: KCl_α and KCl_γ.

Coordination mode<br>Interaction	KCl_α<br>E^2	KCl_γ<br>E^2
$K^+\cdots N_{trz}1$	0.3	4.7
$K^+\cdots N_{trz}2$	0.2	0.1
C_{trz}-I1$\cdots Cl^-$	12.8	21.1
C_{trz}-I2$\cdots Cl^-$	14.6	6.9
C_{trz}-H1$\cdots Cl^-$	2.0	0.0
C_{trz}-H2$\cdots Cl^-$	0.2	4.4
$K^+\cdots Cl^-$	15.7	0.3

Table S6. Distances (Å) and angles (°) for the interactions between K^+ and the N_{trz} atoms, for the halogen and hydrogen bonding interactions with Cl^-, as well as for $K^+\cdots Cl^-$, in the methanol and acetonitrile DFT-optimised **3**·KCl complexes. Data are shown for two coordination modes: KCl_α and KCl_γ.

Coordination mode	KCl_α				KCl_γ			
Solvent	Methanol		Acetonitrile		Methanol		Acetonitrile	
Interaction	Distance	Angle	Distance	Angle	Distance	Angle	Distance	Angle
$K^+\cdots N_{trz}1$	4.97	–	4.97	–	2.89	–	2.89	–
$K^+\cdots N_{trz}2$	5.80	–	5.80	–	5.80	–	5.80	–
C_{trz}-I1$\cdots Cl^-$	3.19	163.71	3.19	163.69	3.13	176.20	3.13	176.21
C_{trz}-I2$\cdots Cl^-$	3.13	173.20	3.13	173.19	3.25	160.86	3.25	160.94
C_{trz}-H1$\cdots Cl^-$	2.74	135.42	2.74	135.41	8.47	23.22	8.47	23.25
C_{trz}-H2$\cdots Cl^-$	3.59	163.87	3.59	163.79	2.76	151.08	2.77	151.14
$K^+\cdots Cl^-$	4.97	–	4.97	–	2.89	–	2.89	–

Table S7. Distances (Å), angles (°), and second-order perturbation interaction energies (E^2, kcal mol^{-1}) for the halogen and hydrogen bonding interactions with Cl^-, as well as for O-H$\cdots Cl^-$, in the gas-phase DFT-optimised **3**·dopamine·HCl binding arrangements. Data are shown for four coordination modes: D_α, D_β, D_γ, and D_δ.

Coordination mode	D_α			D_β			D_γ			D_δ		
Interaction	Distance	Angle	E^2	Distance	Angle	E^2	Distance	Angle	E^2	Distance	Angle	E^2
C_{trz}-I1$\cdots Cl^-$	3.41	157.0	4.8	3.22	168.2	9.8	3.21	177.8	10.0	3.19	166.0	10.2
C_{trz}-I2$\cdots Cl^-$	3.14	172.2	10.9	3.28	175.0	7.9	3.34	170.6	6.2	3.56	153.9	2.8
C_{trz}-H1$\cdots Cl^-$	5.32	121.7	–	9.76	38.2	–	4.20	149.2	–	9.20	39.4	–
C_{trz}-H2$\cdots Cl^-$	3.78	148.7	0.1	4.39	159.5	–	4.85	152.7	–	2.66	138.8	2.7
O-H1$\cdots Cl^-$	2.05	163.9	27.6	2.02	172.4	27.7	2.07	165.6	23.8	2.16	172.9	18.1
O-H2$\cdots Cl^-$	2.24	157.5	12.6	2.06	175.1	26.5	2.29	152.7	9.0	5.19	119.3	0.0

Table S8. Distances (Å) and angles (°) for the halogen and hydrogen bonding interactions with Cl^-, as well as for O-H$\cdots Cl^-$, in the acetone DFT-optimised **3**·dopamine·HCl binding arrangements. Data are shown for four coordination modes: D_α, D_β, D_γ, and D_δ.

Coordination mode	D_α		D_β		D_γ		D_δ	
Interaction	Distance	Angle	Distance	Angle	Distance	Angle	Distance	Angle
C_{trz}-I1$\cdots Cl^-$	3.32	161.8	3.39	162.4	3.28	173.1	3.24	157.0
C_{trz}-I2$\cdots Cl^-$	3.19	175.8	3.30	177.3	3.36	175.0	3.40	156.0
C_{trz}-H1$\cdots Cl^-$	5.33	126.0	9.96	36.5	4.54	153.5	9.25	37.4
C_{trz}-H2$\cdots Cl^-$	4.18	155.2	4.49	167.7	4.98	159.1	2.63	142.9
O-H1$\cdots Cl^-$	2.13	168.5	2.11	170.1	2.15	166.6	2.15	175.5
O-H2$\cdots Cl^-$	2.23	166.2	2.14	173.3	2.20	160.9	5.22	120.1

Table S9. Uncorrected electronic energy differences ($\Delta\varepsilon_0$),[a] zero-point corrections (ΔZPE),[b] and thermal corrected energy differences (ΔE)[c] (all in kcal mol^{-1}) for selected alternative arrangements of **3**·dopamine·HCl.

Condition	Acetone		
Energy term	D_β-D_α	D_β-D_γ	D_β-D_δ
$\Delta\varepsilon_0$	-2.2	-8.9	1.7
ΔZPE	-0.9	-0.6	-0.7
ΔE	-2.9	-9.4	1.0

a) Energy values were not corrected for basis set superposition errors; b) ΔZPE is included in the ΔE term; c) $\Delta E = \Delta\varepsilon_0 + \Delta E_{Tot}$, where ΔE_{Tot} accounts for internal energy differences due to translational, rotational, vibrational, and electronic motions. Absolute energy terms are defined above.

Computational Methods/Molecular Modelling details

General

The theoretical studies reported in this work were conducted using a workflow that included conformational analyses in gas-phase using molecular dynamics (MD) simulations, quantum mechanics (QM) calculations, and MD simulations in solution. The QM calculations, including the derivatisation of restrained electrostatic potential (RESP) atomic charges, were carried out with Gaussian 16.[6] The classical force field-based calculations were performed with AMBER 20.[5]

The starting structure of **3** was generated assembling fragments obtained by manipulation of single crystal X-ray structures deposited with the Cambridge Crystallographic Data Centre (CCDC)[7] under the Cambridge Structural Database (CSD) refcodes WOFBOK (aza-BODIPY dye motif),[8] PIZPOE (proto-iodo-triazole decorated phenyl fragment),[9] and BEXNAT (diaza-18-crown-6 moiety).[10] This structure was optimised using the B3LYP/def2-SVP theory level, combined with Grimme's GD3 empirical dispersion.[11] Afterwards, it was used in the conformational analyses of KCl and dopamine·HCl ion-pair complexes (see below) and in the initial RESP charges calculations.

The dopaminium substrate and $BAr^F{}_4{}^-$ anion were taken from the single crystal X-ray structures with CSD refcodes ATOLIF[12] and ZOPJIB,[13] respectively.

Classical force field calculations

The MM calculations and MD simulations were performed using force field parameters taken from the second generation of the Generalised Amber Force Field (GAFF2),[14] except those involving the boron centre, coupled with RESP atomic charges derivatised as detailed below.[15]

In the MD simulation studies carried out in solution, the methanol, acetonitrile, and acetone solvent molecules were also described with GAFF2 parameters and RESP charges,[14, 15] while Cl^- and K^+ were respectively described with discrete charges of -1 and +1 and van der Waals parameters developed for the TIP3P water model.[16] Due to the lack of bonding and angle parameters for boron in GAFF2, it was necessary to develop suitable parameters for this atomic centre as follows.

Boron force field parameters

The boron bonding terms were derived using VFFDT 1.0.[17] This approach has been successfully used in the parametrisation of boronate derivatives,[18, 19] as well as in boron functionalised guanine-hyaluronic-based polymers.[20] The boron van der Waals parameters were directly taken from reference [21]. The bonding and angle bending terms for **3** and $BArF_4^-$ were calculated with the structures presented in Figure S17a and Figure S18a, optimised at the B3LYP/6-31G(d) theory level using the Seminario method, as implemented in VFFDT 1.0.[17] The values of the ideal bond length and bond angle terms, together with corresponding force constants are listed in Table S10, while the pertinent atom types are given in Figure S17b and Figure S18b. The dihedral angle and improper terms were adapted from GAFF2 considering that boron is equivalent to a tetrahedral c3 carbon atom type, as summarised in Table S10.

Table S10. Boron force field parameters used in this work.

**Mass**	**Atomic weight (A_r)**				**Notes**
B	10.81				Taken from ref. [22]
**Bond lengths**	**K_r (kcal mol^{-1} Å^{-2})**	**r_{eq} (Å)**			**Notes**
f -B	268.050	1.393			Determined with VFFDT
na-B	137.925	1.559			Determined with VFFDT
ca-B	127.258	1.656			Determined with VFFDT
**Bond angles**	**K_θ (kcal mol^{-1} rad^{-2})**	**ϑ_{eq} (°)**			**Notes**
f -B -f	63.641	110.501			Determined with VFFDT
f -B -na	94.853	110.032			Determined with VFFDT
na-B -na	135.231	106.124			Determined with VFFDT
ca-B -ca	121.047	109.479			Determined with VFFDT
ca-ca-B	134.449	122.209			Determined with VFFDT
**Dihedral angles**	**Scaling factor**	**$V_n/2$ (kcal mol^{-1})**	**γ (°)**	**periodicity *N***	**Notes**
f -B -na-cc	1	0.000	0.000	1.0	GAFF2 parameters for f -c3-na-cc
f -B -na-cd	1	0.000	0.000	1.0	GAFF2 parameters for f -c3-na-cd
na-B -na-cd	1	0.000	0.000	1.0	GAFF2 parameters for na-c3-na-cd
na-B -na-cc	1	0.000	0.000	1.0	GAFF2 parameters for na-c3-na-cc
cc-cc-na-B	1	1.700	180.000	2.0	GAFF2 parameters for cc-cc-na-c3
cd-cd-na-B	1	1.700	180.000	2.0	GAFF2 parameters for cd-cd-na-c3
ce-cd-na-B	1	1.700	180.000	2.0	GAFF2 parameters for ce-cd-na-c3
c3-cc-na-B	1	1.700	180.000	2.0	GAFF2 parameters for c3-cc-na-c3
B -ca-ca-ca	4	14.500	180.000	2.0	GAFF2 parameters for c3-ca-ca-ca
ca-B -ca-ca	1	0.000	180.000	2.0	GAFF2 parameters for ca-c3-ca-ca
**Improper dihedral**		**K_Φ (kcal mol-1)**	**Φ_0 (°)**	**periodicity *N***	**Notes**
B -cc-na-cd		1.100	180.000	2.0	GAFF2 parameters for c3-cc-na-cd
B -ca-ca-ca		1.100	180.000	2.0	GAFF2 parameters for c3-ca-ca-ca
**Van der Waals**	**r (Å)**	**ε (kcal mol^{-1})**			**Notes**
B	1.980	0.034			Taken from ref. [21]

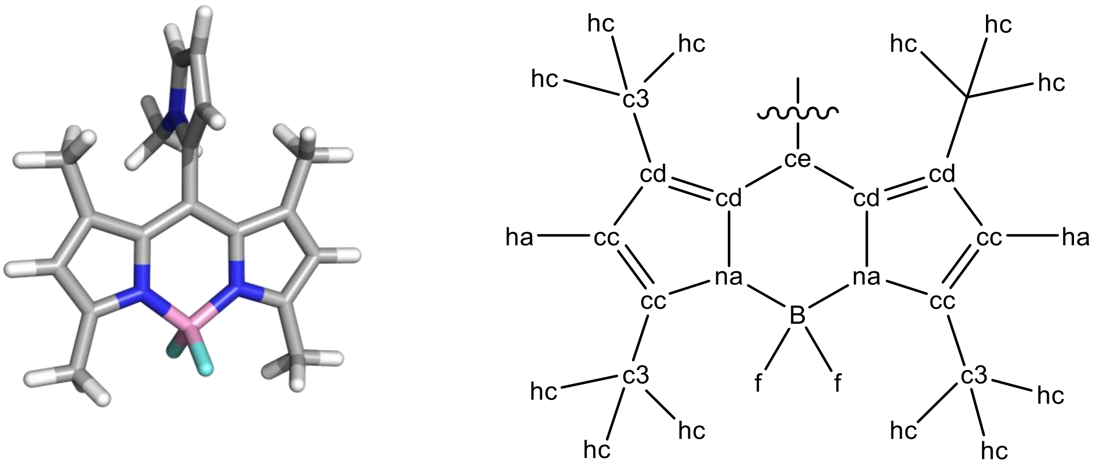

a) b)

Figure S17. a) B3LYP/6-31G(d) optimised structure of BODIPY model compound (WOFBOK[8]); b) GAFF2 and custom atom types applied to this molecule and **3**.

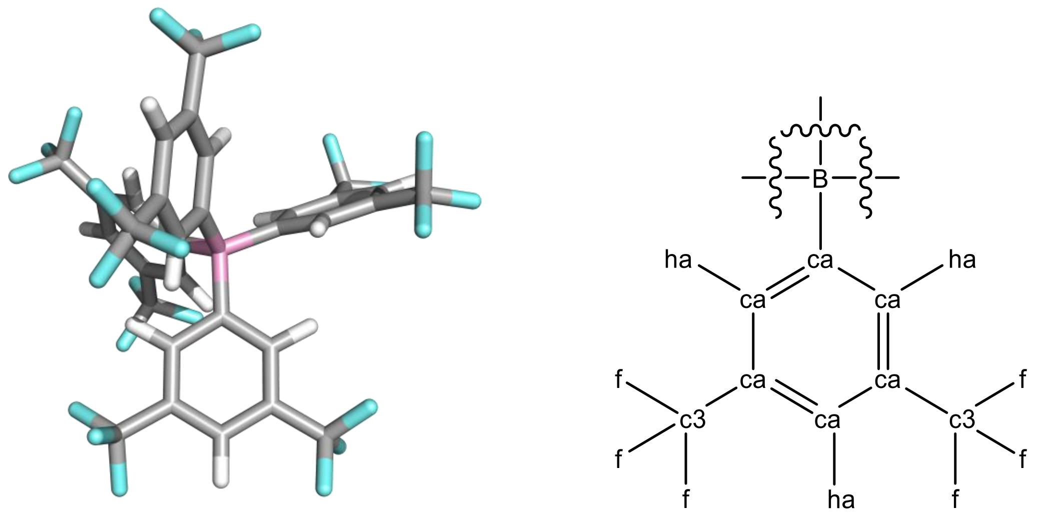

a) b)

Figure S18. a) B3LYP/6-31G(d) optimised structure of $BAr^F{}_4^–$ (ZOPJIB[13]); b) GAFF2 and custom atom types applied.

RESP atomic charges and extra point of charge derivatisation

In agreement with the charge derivatisation philosophy for GAFF,[14] the XB-based host **3**, the dopaminium guest, the $BAr^F{}_4^–$ counter-ion, and the methanol, acetonitrile, and acetone solvent molecules were optimised at the HF/6-31G(d) theory level. The iodine atoms in **3** were described with the 6-311G(d) basis set. Subsequently, the atomic charges were obtained in two RESP charge fitting stages using the electrostatic potential estimated at the same level of theory.

In the MD simulations performed in solution, following our previous modelling investigations on anion recognition by halogen bonds using a classical MD approach,[23-30] the two putative XB interactions between **3** and $Cl^–$ were described by the addition of a massless extra point (EP) of charge to GAFF2.[31] Thus, an EP with a van der Waals parameter and mass set to zero was positioned in front of each iodine binding unit to mimic its σ-hole. The parameterisation of the EP bonding parameter and atomic partial charge was performed using the **3**·KCl complex wrapped binding arrangement previously DFT optimised in the gas-phase at the M06-2X/def2-TZVP(D) theory level. Several I−EP distances, from 2.00 to 3.50 Å with increments of 0.01 Å, were systematically evaluated in gas-phase by MM through the energy minimisations of the **3**·KCl association. The different I−EP distances were fixed using a high bond stretching force constant of 600 kcal $mol^{–1}$ $Å^{–2}$, while an angle bending force constant of 150 kcal $mol^{–1}$ $rad^{–2}$ was used for the C–I–EP ideal angle of 180°. For a given I−EP distance,

the RESP atomic charges were re-calculated, as follows: in the structure **3**, an EP was linearly positioned in front of each C−I bond at the desired I−EP distance. Subsequently the corresponding atomic charges were calculated via a two-stage RESP fitting of the HF obtained electrostatic potential. Out of the tens of I−EP distances tested, it was found that the distance of 2.64 Å, along with the RESP charges summarised in Table S11, enabled the gas-phase MM optimisation of the KCl ion-pair complex of **3**. This structure displays two XB interactions with I···Cl⁻ distances of 3.17 and 3.36 Å and C–I···Cl⁻ angles of 176.0 and 176.3°, comparable to the ones estimated by DFT. These EP parameters allowed us to undertake the subsequent MD simulations in solution with the KCl and dopamine·HCl ion-pair complexes (see below).

Table S11. Iodine and EP RESP charges (*e*) in 3 for the selected I–EP distances (Å).

Receptor	I–EP distances	I	EP
**3**	no EP	0.034677	–
	2.64	-0.129276	0.035424

Conformational Analyses

To explore the possible conformations adopted by **3** upon KCl and dopamine·HCl ion pairs' recognition, conformational analysis runs were carried out, using the initial RESP atomic charges with two putative XB interactions maintained through I···Cl⁻ distance and C–I···Cl⁻ angle restraints. Moreover, additional distance and angles restraints on the O–H···Cl⁻ hydrogen bonding interactions between **3** and the dopamine·HCl substrate were applied, along with NH_3^+···O distances restraints between the dopaminium's protonated amine group and **3**'s aza-crown moiety. The different associations were then subject to the following protocol: An initial structure was minimised by molecular mechanics and subsequently heated in gas phase to 500 K for 50 ps, followed by a collection run of 1 or 3 ns, using a time step of 1 fs. Frames were saved every 0.1 ps leading to trajectory file containing 10000 or 30000 structures. The high temperature ensured that conformational barriers were surmounted, enabling a broader coverage of the conformational space. Afterwards, all these structures were minimised by MM using a steepest descendent gradient followed by the conjugate gradient algorithm, until the convergence criterium of 0.0001 kcal mol^{-1} $Å^{-1}$ was attained. The optimised structures were then RMSD clustered using UCSF Chimera 1.17.[32] Two representative structures of **3** associated with KCl displaying almost linear XB interactions

and different conformational binding arrangements (KCl_α and KCl_β) were selected for subsequent DFT calculations. An equivalent approach was used to obtain the D_α, D_β, and D_γ alternative binding arrangements, which mainly differ in the relative spatial disposition adopted by the dopaminium ligand relatively to **3**, as described in the main text.

Quantum Calculations on K^+ and ion-pair complexes of **3**

After the gas-phase conformational analyses, the selected binding arrangements for KCl and dopamine·HCl were optimised by DFT method using the M06-2X functional coupled with def2-TZVP basis set, while the halogens were described with the def2-TZVPD basis set. Furthermore, these DFT calculations were further extended to the KCl_γ and D_δ binding scenarios, respectively taken from the MD simulations carried with **3**·KCl in acetonitrile/methanol 1:1 (*v*/*v*) solvent mixture and **3**·dopamine·HCl in acetone (see below).

All DFT calculations performed with **3'**·K^+ model complex, the truncated version of **3** without the XB-BODIPY-appended arms, were also performed at the M06-2X/DEF2TZVP theory level.

DFT calculations performed in gas-phase were followed by geometry optimisations in acetonitrile, methanol, or acetone at the same level of theory. The solvent effects in the calculations were considered using the Polarisable Continuum Model (PCM).[33, 34]

The absence of imaginary frequencies on the vibrational frequency analyses of all geometry optimised structures confirmed that all stationary point geometries were local minima on the potential energy surface. Further analyses of the XB interactions on the optimised associations were carried out in gas-phase with Natural Bond Orbital approach,[35] using NBO7.[36, 37]

Molecular dynamics simulations

The K^+ and dopamine·HCl complexes of **3** were investigated by MD simulations in acetonitrile/methanol 1:1 (*v*/*v*) solvent mixture as well as in acetone, accordingly with the experimental binding studies. The MD simulations with **3**·KCl ion-pair complex were only undertaken in the solvent mixture. Thus, the structures obtained by DFT calculations were solvated in cubic boxes using PackMOL.[38] The 1:1 volume ratio of the solvent mixture was achieved with 803 MeOH and 623 MeCN molecules for the K^+ complex, whilst for the KCl and

dopamine·HCl ion-pair complexes 928 MeOH and 720 MeCN molecules were used. The acetone cubic boxes built for K^+ and dopamine·HCl complexes contained 886 solvent molecules. Moreover, the solvated systems with **3**·K^+ were charge neutralised with the addition of a $BAr^F{}_4{}^-$ counterion. While **3**·KCl was simulated starting from the wrapped KCl_α conformational binding arrangement, the **3**·dopamine·HCl complex was evaluated using the D_α, D_β, and D_γ binding conformations as starting scenarios. Regardless of the number of starting points, three MD runs were performed for each system, which were equilibrated under periodic boundary conditions using the following multistage protocol. The system was relaxed by MM minimisation of solvent molecules and by keeping the solutes fixed with a positional restraint of 500 kcal mol^{-1} Å^{-2}. The restraint was then removed, allowing the entire system to relax. These two minimisation stages comprised an initial set of 10000 steepest descent algorithm steps, followed by 10000 steps of conjugated gradient algorithm. The equilibration proceeded with heating up the system to 300 K for 100 ps using a NVT ensemble and a weak positional restraint (10 kcal mol^{-1} Å^{-2}) on the solutes. Afterwards, each system's density was allowed to equilibrate in an NPT ensemble at 1 atm for 1.0 ns, at the same temperature, with a weaker positional restraint (5 kcal mol^{-1} Å^{-2}) on the solutes. The positional restraint was removed in a short 100 ps run, which was followed by an NPT data collection run of 50 ns (**3**·KCl and **3**·dopamine·HCl) or 100 ns (**3**·K^+). The collection run's trajectory frames were saved every 10.0 ps. Three independent runs were performed for each system. The CUDA version of the PMEMD executable was used for the simulation of all solvated systems.[39, 40] The bond lengths involving all hydrogen atoms were constrained with the SHAKE algorithm, allowing the usage of a 2.0 fs time step.[41] The Particle Mesh Ewald (PME) method was used to treat the long-range electrostatic interactions.[42] The non-bonded van der Waals interactions were truncated with a 10 Å cut-off. The temperature of the system was maintained independently by coupling the system to an external bath temperature of 300 K, using Langevin dynamics,[43] with a collision frequency γ of 1.0 ps^{-1}. The pressure was controlled by the Berendsen barostat[44] at 1 atm and compressibility of 44.6×10^{-6} bar^{-1}, with a relaxation time of 1.0 ps. The post-processing of the MD trajectory files to obtain structural data was performed with cpptraj.[45]

Notes and References

1. OriginLab Corporation, OriginPro, 2023.
2. K. A. Arnold, A. M. Viscariello, M. Kim, R. D. Gandour, F. R. Fronczek and G. W. Gokel, N,N'-Bis(substituted)-4,13-diaza-18-crown-6 derivatives having pi-donor-group-sidearms: correlation of thermodynamics and solid state structures, *Tetrahedron Lett.*, 1988, **29**, 3025-3028.
3. J. Hu and G. W. Gokel, Solution complexation between potassium iodide and lariat ethers having pi-donor sidearms, *Chem. Commun.*, 2003, 2536-2537.
4. A. J. Taylor, R. Hein, S. C. Patrick, J. J. Davis and P. D. Beer, Anion Sensing through Redox-Modulated Fluorescent Halogen Bonding and Hydrogen Bonding Hosts, *Angew. Chem. Int. Ed.*, 2024, **63**, e202315959.
5. D. A. Case, K. Belfon, I. Y. Ben-Shalom, S. R. Brozell, D. S. Cerutti, I. T.E. Cheatham, V. W. D. Cruzeiro, T. A. Darden, R. E. Duke, G. Giambasu, M. K. Gilson, H. Gohlke, A. W. Goetz, R. Harris, S. Izadi, S. A. Izmailov, K. Kasavajhala, A. Kovalenko, R. Krasny, T. Kurtzman, T. S. Lee, S. LeGrand, P. Li, C. Lin, J. Liu, T. Luchko, R. Luo, V. Man, K. M. Merz, Y. Miao, O. Mikhailovskii, G. Monard, H. Nguyen, A. Onufriev, F. Pan, S. Pantano, R. Qi, D. R. Roe, A. Roitberg, C. Sagui, S. Schott-Verdugo, J. Shen, C. L. Simmerling, N. R. Skrynnikov, J. Smith, J. Swails, R. C. Walker, J. Wang, L. Wilson, R. M. Wolf, X. Wu, Y. Xiong, Y. Xue, D. M. York and P. A. Kollman, AMBER 20., 2020.
6. M. J. Frisch, G. W. Trucks, H. B. Schlegel, G. E. Scuseria, M. A. Robb, J. R. Cheeseman, G. Scalmani, V. Barone, G. A. Petersson, H. Nakatsuji, X. Li, M. Caricato, A. V. Marenich, J. Bloino, B. G. Janesko, R. Gomperts, B. Mennucci, H. P. Hratchian, J. V. Ortiz, A. F. Izmaylov, J. L. Sonnenberg, D. Williams-Young, F. Ding, F. Lipparini, F. Egidi, J. Goings, B. Peng, A. Petrone, T. Henderson, D. Ranasinghe, V. G. Zakrzewski, J. Gao, N. Rega, G. Zheng, W. Liang, M. Hada, M. Ehara, K. Toyota, R. Fukuda, J. Hasegawa, M. Ishida, T. Nakajima, Y. Honda, O. Kitao, H. Nakai, T. Vreven, K. Throssell, J. J. A. Montgomery, J. E. Peralta, F. Ogliaro, M. J. Bearpark, J. J. Heyd, E. N. Brothers, K. N. Kudin, V. N. Staroverov, T. A. Keith, R. Kobayashi, J. Normand, K. Raghavachari, A. P. Rendell, J. C. Burant, S. S. Iyengar, J. Tomasi, M. Cossi, J. M. Millam, M. Klene, C. Adamo, R. Cammi, J. W. Ochterski, R. L. Martin, K. Morokuma, O. Farkas, J. B. Foresman and D. J. Fox, Gaussian 16, Revision C.01., 2019.
7. C. R. Groom, I. J. Bruno, M. P. Lightfoot and S. C. Ward, The Cambridge Structural Database, *Acta Crystallogr. B*, 2016, **72**, 171-179.
8. H. Wang, M. G. Vicente, F. R. Fronczek and K. M. Smith, Synthesis and transformations of 5-chloro-2,2'-dipyrrins and their boron complexes, 8-chloro-BODIPYs, *Chem. Eur. J.*, 2014, **20**, 5064-5074.
9. H. Juwarker, J. M. Lenhardt, D. M. Pham and S. L. Craig, 1,2,3-Triazole CH...Cl(-) contacts guide anion binding and concomitant folding in 1,4-diaryl triazole oligomers, *Angew. Chem. Int. Ed.*, 2008, **47**, 3740-3743.
10. C. Pascard, C. Riche, M. Cesario, F. Kotzyba-Hibert and J. M. Lehn, Coreceptor–substrate binding. Crystal structures of a macrotricyclic ligand and of its molecular cryptate with the cadaverine dication, *Chem. Commun.*, 1982, 557-560.
11. S. Grimme, J. Antony, S. Ehrlich and H. Krieg, A consistent and accurate ab initio parametrization of density functional dispersion correction (DFT-D) for the 94 elements H-Pu, *J. Chem. Phys.*, 2010, **132**, 154104.

12. B. Ivanova and M. Spiteller, Salts of aromatic amines: crystal structures, spectroscopic and non-linear optical properties, *Spectrochimica Acta. A*, 2010, **77**, 849-855.

13. L. E. McNamara, J. N. Boyn, S. W. Anferov, A. S. Filatov, M. W. Maloney, D. A. Mazziotti, R. D. Schaller and J. S. Anderson, Variable Peripheral Ligand Donation Tunes Electronic Structure and NIR II Emission in Tetrathiafulvalene Tetrathiolate Diradicaloids, *J. Am. Chem. Soc.*, 2024, **146**, 17285-17295.

14. J. Wang, R. M. Wolf, J. W. Caldwell, P. A. Kollman and D. A. Case, Development and testing of a general amber force field, *J. Comput. Chem.*, 2004, **25**, 1157-1174.

15. C. I. Bayly, P. Cieplak, W. Cornell and P. A. Kollman, A well-behaved electrostatic potential based method using charge restraints for deriving atomic charges: the RESP model, *J. Phys. Chem.*, 2002, **97**, 10269-10280.

16. P. Li, L. F. Song and K. M. Merz, Jr., Systematic Parameterization of Monovalent Ions Employing the Nonbonded Model, *J. Chem. Theory. Comput.*, 2015, **11**, 1645-1657.

17. S. Zheng, Q. Tang, J. He, S. Du, S. Xu, C. Wang, Y. Xu and F. Lin, VFFDT: A New Software for Preparing AMBER Force Field Parameters for Metal-Containing Molecular Systems, *J. Chem. Theory Comput.*, 2016, **56**, 811-818.

18. B. Kurt and H. Temel, Parameterization of Boronates Using VFFDT and Paramfit for Molecular Dynamics Simulation, *Molecules*, 2020, **25**, 2196.

19. B. Kurt and H. Temel, Development of AMBER parameters for molecular dynamics simulations of boron compounds containing aromatic structure, *Chem. Phys. Lett.*, 2021, **775**.

20. V. Sousa, A. J. R. Amaral, E. J. Castanheira, I. Marques, J. M. M. Rodrigues, V. Felix, J. Borges and J. F. Mano, Self-Supporting Hyaluronic Acid-Functionalized G-Quadruplex-Based Perfusable Multicomponent Hydrogels Embedded in Photo-Cross-Linkable Matrices for Bioapplications, *Biomacromolecules*, 2023, **24**, 3380-3396.

21. D. S. Otkidach and I. V. Pletnev, Conformational analysis of boron-containing compounds using Gillespie–Kepert version of molecular mechanics, *J. Mol. Struct. THEOCHEM*, 2001, **536**, 65-72.

22. T. Prohaska, J. Irrgeher, J. Benefield, J. K. Böhlke, L. A. Chesson, T. B. Coplen, T. Ding, P. J. H. Dunn, M. Gröning, N. E. Holden, H. A. J. Meijer, H. Moossen, A. Possolo, Y. Takahashi, J. Vogl, T. Walczyk, J. Wang, M. E. Wieser, S. Yoneda, X.-K. Zhu and J. Meija, Standard atomic weights of the elements 2021 (IUPAC Technical Report), *Pure Appl. Chem.*, 2022, **94**, 573-600.

23. M. J. Langton, S. W. Robinson, I. Marques, V. Felix and P. D. Beer, Halogen bonding in water results in enhanced anion recognition in acyclic and rotaxane hosts, *Nat. Chem.*, 2014, **6**, 1039-1043.

24. T. A. Barendt, A. Docker, I. Marques, V. Felix and P. D. Beer, Selective Nitrate Recognition by a Halogen-Bonding Four-Station [3]Rotaxane Molecular Shuttle, *Angew. Chem. Int. Ed.*, 2016, **55**, 11069-11076.

25. J. Y. C. Lim, I. Marques, L. Ferreira, V. Felix and P. D. Beer, Enhancing the enantioselective recognition and sensing of chiral anions by halogen bonding, *Chem. Commun.*, 2016, **52**, 5527-5530.

26. J. Y. C. Lim, I. Marques, V. Felix and P. D. Beer, Enantioselective Anion Recognition by Chiral Halogen-Bonding [2]Rotaxanes, *J. Am. Chem. Soc.*, 2017, **139**, 12228-12239.

27. J. Y. C. Lim, I. Marques, V. Felix and P. D. Beer, A Chiral Halogen-Bonding [3]Rotaxane for the Recognition and Sensing of Biologically Relevant Dicarboxylate Anions, *Angew. Chem. Int. Ed.*, 2018, **57**, 584-588.

28. J. Y. C. Lim, I. Marques, V. Felix and P. D. Beer, Chiral halogen and chalcogen bonding receptors for discrimination of stereo- and geometric dicarboxylate isomers in aqueous media, *Chem. Commun.*, 2018, **54**, 10851-10854.

29. A. Borissov, I. Marques, J. Y. C. Lim, V. Felix, M. D. Smith and P. D. Beer, Anion Recognition in Water by Charge-Neutral Halogen and Chalcogen Bonding Foldamer Receptors, *J. Am. Chem. Soc.*, 2019, **141**, 4119-4129.

30. E. J. Mitchell, A. J. Beecroft, J. Martin, S. Thompson, I. Marques, V. Felix and P. D. Beer, Hydrosulfide (HS(-)) Recognition and Sensing in Water by Halogen Bonding Hosts, *Angew. Chem. Int. Ed.*, 2021, **60**, 24048-24053.

31. M. A. Ibrahim, Molecular mechanical study of halogen bonding in drug discovery, *J. Comput. Chem.*, 2011, **32**, 2564-2574.

32. E. F. Pettersen, T. D. Goddard, C. C. Huang, G. S. Couch, D. M. Greenblatt, E. C. Meng and T. E. Ferrin, UCSF Chimera--a visualization system for exploratory research and analysis, *J. Comput. Chem.*, 2004, **25**, 1605-1612.

33. J. Tomasi, B. Mennucci and R. Cammi, Quantum mechanical continuum solvation models, *Chem. Rev.*, 2005, **105**, 2999-3093.

34. G. Scalmani and M. J. Frisch, Continuous surface charge polarizable continuum models of solvation. I. General formalism, *J. Chem. Phys.*, 2010, **132**, 114110.

35. E. D. Glendening, C. R. Landis and F. Weinhold, Natural bond orbital methods, *Wiley Interdiscip. Rev. Comput. Mol. Sci.*, 2011, **2**, 1-42.

36. E. D. Glendening, K. Badenhoop, A. E. Reed, J. E. Carpenter, J. A. Bohmann, C. M. Morales, P. Karafiloglou, C. R. Landis and F. Weinhold, NBO 7.0., 2018.

37. E. D. Glendening, C. R. Landis and F. Weinhold, NBO 7.0: New vistas in localized and delocalized chemical bonding theory, *J. Comput. Chem.*, 2019, **40**, 2234-2241.

38. L. Martinez, R. Andrade, E. G. Birgin and J. M. Martinez, PACKMOL: a package for building initial configurations for molecular dynamics simulations, *J. Comput. Chem.*, 2009, **30**, 2157-2164.

39. R. Salomon-Ferrer, A. W. Gotz, D. Poole, S. Le Grand and R. C. Walker, Routine Microsecond Molecular Dynamics Simulations with AMBER on GPUs. 2. Explicit Solvent Particle Mesh Ewald, *J. Chem. Theory Comput.*, 2013, **9**, 3878-3888.

40. S. Le Grand, A. W. Götz and R. C. Walker, SPFP: Speed without compromise—A mixed precision model for GPU accelerated molecular dynamics simulations, *Comput. Phys. Commun.*, 2013, **184**, 374-380.

41. J.-P. Ryckaert, G. Ciccotti and H. J. C. Berendsen, Numerical integration of the cartesian equations of motion of a system with constraints: molecular dynamics of n-alkanes, *J. Comput. Phys.*, 1977, **23**, 327-341.

42. T. Darden, D. York and L. Pedersen, Particle mesh Ewald: An N·log(N) method for Ewald sums in large systems, *J. Chem. Phys.*, 1993, **98**, 10089-10092.

43. R. J. Loncharich, B. R. Brooks and R. W. Pastor, Langevin dynamics of peptides: the frictional dependence of isomerization rates of N-acetylalanyl-N'-methylamide, *Biopolymers*, 1992, **32**, 523-535.

44. H. J. C. Berendsen, J. P. M. Postma, W. F. van Gunsteren, A. DiNola and J. R. Haak, Molecular dynamics with coupling to an external bath, *J. Chem. Phys.*, 1984, **81**, 3684-3690.

45. D. R. Roe and T. E. Cheatham, 3rd, PTRAJ and CPPTRAJ: Software for Processing and Analysis of Molecular Dynamics Trajectory Data, *J. Chem. Theory Comput.*, 2013, **9**, 3084-3095.
